# Genetic and molecular mechanisms underlying the *parthenocarpic fruit* mutation in tomato

**DOI:** 10.3389/fpls.2024.1329949

**Published:** 2024-03-27

**Authors:** Maurizio E. Picarella, Fabrizio Ruiu, Luigi Selleri, Silvia Presa, Chiara Mizzotti, Simona Masiero, Lucia Colombo, Gian Piero Soressi, Antonio Granell, Andrea Mazzucato

**Affiliations:** ^1^ Dipartimento di Scienze Agrarie e Forestali (DAFNE), Università degli Studi della Tuscia, Viterbo, Italy; ^2^ Departamento de Biotecnología de Cultivos, Instituto de Biología Molecular y Celular de Plantas (IBMCP), Consejo Superior de Investigaciones Científicas (CSIC) – Universitat Politécnica de Valéncia (UPV), Valencia, Spain; ^3^ Dipartimento di Bioscienze (DBS), Università degli Studi di Milano, Milano, Italy

**Keywords:** fruit set, HD-Zip III transcription factors, ovule development, parthenocarpy, seedlessness

## Abstract

Parthenocarpy allows fruit set independently of fertilization. In parthenocarpic-prone tomato genotypes, fruit set can be achieved under pollen-limiting environmental conditions and in sterile mutants. Parthenocarpy is also regarded as a quality-related trait, when seedlessness is associated with positive fruit quality aspects. Among the different sources of genetic parthenocarpy described in tomato, the *parthenocarpic fruit* (*pat*) mutation is of particular interest because of its strong expressivity, high fruit set, and enhanced fruit quality. The complexity of the *pat* “syndrome” associates a strong competence for parthenocarpy with a complex floral phenotype involving stamen and ovule developmental aberrations. To understand the genetic basis of the phenotype, we mapped the *pat* locus within a 0.19-cM window of Chr3, comprising nine coding loci. A non-tolerated missense mutation found in the 14^th^ exon of *Solyc03g120910*, the tomato ortholog of the *Arabidopsis* HD-Zip III transcription factor *HB15* (*SlHB15*), cosegregated with the *pat* phenotype. The role of SlHB15 in tomato reproductive development was supported by its expression in developing ovules. The link between *pat* and *SlHB15* was validated by complementation and knock out experiments by co-suppression and CRISPR/Cas9 approaches. Comparing the phenotypes of *pat* and those of *Arabidopsis HB15* mutants, we argued that the gene plays similar functions in species with fleshy and dry fruits, supporting a conserved mechanism of fruit set regulation in plants.

## 1 Introduction

The fruit forms through an intimate developmental collaboration between ovules (the seeds precursors) and carpels (the fruit precursors). The ovary, which develops in concert with the rest of the flower organs (Phase I, Gillaspy et al., 1993), ceases to undergo cell divisions before anthesis and enters a “growth arrest” state. Pollination and subsequent fertilization require pollen germination, penetration, and growth of the pollen tube in the stylar tissue towards the ovule, the structure containing the gametophyte, to fuse with the egg cell (Dumas and Mogensen, 1993). Only after fertilization is successfully completed, a signal produced by the young embryo provokes the ovary to resume growth. This developmental decision is referred to as “fruit set”. Only then fruit development starts involving an initial phase of rapid cell division lasting in tomato (*Solanum lycopersicum* L.) 5–10 days (Phase II), while the subsequent growth is mainly driven by cell enlargement (Phase III, Gillaspy et al., 1993). Once reached its full size (mature green), the fruit enters the ripening processes.

Fruit set may occasionally be independent of pollination and fertilization, a process known as parthenocarpy (Shinozaki and Ezura, 2016). Parthenocarpy occurs spontaneously but it can also be induced and, for a long time, humans have attempted to develop seedless fruits with the application of various hormones or by selecting some mutations (Gustafson, 1942; Schwabe and Mills, 1981). Parthenocarpy represents an interesting commodity, because seedless fruits are easier to consume and develop without the environmental and ecological constraints of the reproductive process (Picarella and Mazzucato, 2019). Despite the relative abundance of natural genetic sources of parthenocarpy identified in different species, few of the underlying genes have been identified so far. In apple cultivars that produce flowers lacking petals and stamens, parthenocarpy is based on a loss-of-function mutation of the class-B MADS-box gene ortholog to *PISTILLATA* (*MdPI*, Yao et al., 2001). Other mutations of genes involved in stamen identity were responsible for occasional (accidental) parthenocarpy in male sterile tomato mutants, such as *stamenless* (*sl*, Gómez et al., 1999) and *pistillate* (*pi*, Olimpieri and Mazzucato, 2008). In *Arabidopsis*, *Auxin Response Factor8* (*ARF8*) underlies parthenocarpy in the *fruit without fertilization* (*fwf*) mutant (Goetz et al., 2006). Introduction of the *fwf* allele in tomato also resulted in parthenocarpy, indicating that the tomato ortholog, *SlARF8*, also plays a role in fruit set (Goetz et al., 2007). Another gene identified as responsible for seedless fruit production was identified in a sugar apple (*Annona squamosa* L.) mutant that fails to form seeds for a defect in ovule development (Lora et al., 2011). In this genotype, the ovules lack the outer integument, due to the mutation of the ortholog of the *Arabidopsis* gene *Inner no outer* (Lora et al., 2011).

In tomato, genes related to the action of auxin have been shown to have an important role in fruit set as demonstrated by several reverse genetics approaches. These include *SlIAA9* (Wang et al., 2005; Saito et al., 2011; Mazzucato et al., 2015) that mediates the indole-3-acetic acid (IAA)-inductive signal for ARF members, *SlARF7* (de Jong et al., 2009) and *SlARF5* (Liu et al., 2018), acting as a negative regulators of fruit set, *AUXIN CUM SILENCING ACTION* (*AUCSIA1* and *AUCSIA2*), that encode small polypeptides involved in either auxin synthesis or transport (Molesini et al., 2009), *PIN-FORMED* (*PIN*, Mounet et al., 2012), an auxin efflux transporter, *SlTIR1*, a putative auxin receptor (Ren et al., 2011) and the transcriptional co-repressor *SlTPL1* (He et al., 2021).

In addition to auxin-related genes, key actors in the tomato fruit set include those controlling gibberellin (GA) metabolism and function. The transcription of members of the tomato GA20ox family, which mediates bioactive GA synthesis, increases in ovaries after pollination and after parthenocarpic setting and over expression (OE) of these genes by transgenesis can lead to parthenocarpy (Olimpieri et al., 2007; Serrani et al., 2007). Engineered tomato mutants in the GA response repressor *DELLA* gene (*SlDELLA*, Martí et al., 2007), also represented by the spontaneous loss-of-function mutant *procera* (*pro*; Bassel et al., 2008), show parthenocarpic fruit development. In addition to auxin and GAs, genes involved in the action of other hormones, such as cytochinins (Matsuo et al., 2012) and ethylene (Lin et al., 2008), as well as genes related to flower development (Pnueli et al., 1994; Ampomah-Dwamena et al., 2002; de Martino et al., 2006; Klap et al., 2017) and to flavonoid metabolism (Schijlen et al., 2007; Ingrosso et al., 2011) have been involved in tomato fruit set.

Several parthenocarpic mutants, spontaneous or induced by mutagenesis or by broad genetic crosses, have been described in tomato (Picarella and Mazzucato, 2019). Recently, the identity of *hydra*, a floral mutant occasionally producing small seedless fruits was associated to a defective function of the *SPOROCYTELESS*/*NOZZLE* (*SPL*/*NZZ*) tomato ortholog (Rojas-Gracia et al., 2017).

Here, we focus on the tomato *parthenocarpic fruit* (*pat*) mutation, obtained by ethyl methanesulfonate (EMS) mutagenesis (Bianchi and Soressi, 1969). The *pat* allele induces parthenocarpic development with strong expressivity along with floral pleiotropic phenotypes, such as short stamens and aberrant ovules (Mazzucato et al., 1998). Parthenocarpic *pat* fruits are always about 30% smaller than those of the respective wild type, and parthenocarpy is facultative; according to internal (truss order) or external (temperature, daylength) conditions, seedlessness may be complete or partial (Mazzucato et al., 1998; 1999; 2003). The *pat* phenotype thus suggested that parthenocarpy could be an induced, secondary effect of a mutated gene, whose primary function is to regulate floral organ development (Mazzucato et al., 1998). However, genetic analysis showed that the *pat* gene is not allelic to tomato mutations involved in the MADS-box B function, such as *sl-2* and *pi* (Mazzucato et al., 2008).

Previous research revealed that the *pat* locus is located within a 1.2-cM region in the long arm of Chr3, between the conserved ortholog set (COS; Fulton et al., 2002) markers T0796 and T1143 (Beraldi et al., 2004). Here, we describe the fine mapping of the *pat* gene and the identification of a candidate belonging to the class III HD-Zip homeobox family. Molecular characterization showed that the *pat* syndrome is due to a mutation that compromises its function and negatively affects the transcription of the gene itself. Validation of such candidate was achieved by functional experiments. Finally, the comparison with *Arabidopsis* mutants affected in the ortholog gene supported the hypothesis that the PAT protein may exert similar functions in a species with dry fruit.

## 2 Materials and methods

### 2.1 Plant materials and growth conditions

To finely map the *pat* locus, the BC_1_F_1_ and BC_1_F_2_ populations derived from the interspecific cross between the tomato line homozygous for the *pat* mutation and *S. pennellii* L (Beraldi et al., 2004) were expanded to 625 and 664 individuals, respectively. The *pat-*mutant line used was in the background of cv. Chico III (determinate growth, *sp*/*sp*), and *S. pennellii* was a plant from LA716 (obtained from the C.M. Rick Tomato Genetics Resource Center, TGRC).

To validate the position of markers putatively linked to the target locus, a set of *S. pennellii* alien substitution lines for Chr1, 2, 3, 4, 6, 8, and 11 (TGRC accession numbers LA2091, LA1639, LA1640, LA3469, LA3142, LA1642, and LA1643, respectively; Rick, 1969) and of introgression lines (ILs; Eshed and Zamir, 1995) were used. To compare mutant phenotypes, cv. Chico III was used as a wild-type non-mutant control (WT). All plants were grown in an unheated tunnel under ambient light conditions in Viterbo, Italy (42°260′N, 12°040′E), in late-spring summer. During the flowering period (month of May), the mean natural photoperiod was 14.5h and temperatures ranged between 12°C and 29°C. Experiments involving transgenics or edited plants were carried out in growth chamber at 24 ± 2°C under a 16/8h light/dark photoperiod.

Two *Arabidopsis ATHB15* mutants were also studied: *corona-1* (*cna-1*; Green et al., 2005) and its Col-0 WT background (provided by G. Morelli, CREA-GB, Italy), and *incurvata4-1* (*icu4-1*; Ochando et al., 2006) and its En-2 WT background (provided by J.L. Micol, Universidad Miguel Hernández, Spain). Twenty plants of each genotype were cultured at 24°C ± 2°C in growth chamber under a 16/8h light/dark photoperiod.

### 2.2 Identification of candidate genes and validation

To fine map the *pat* locus, new local markers were obtained exploiting the tomato/*Arabidopsis* microsynteny and mapping bacterial artificial chromosome (BAC) ends (**Supplementary Data S1**). The sequence comprised between COS markers T17 and T20 was finally blasted at the Solanaceae Genomics Network database (SGN; www.sgn.cornell.edu), and a total of nine genes annotated within the target region were identified. To sequence the candidate genes in the WT and *pat* mutant line, specific primers were designed (**Supplementary Table 1**). Total DNA was extracted from young leaf tissue according to Doyle and Doyle (1990). Polymerase chain reaction (PCR) was performed in 25 μl, using 50 ng of genomic DNA, 2.5 μl of 10× PCR buffer, 2 μl of 10 mM dNTPs, 1.5 μl of 25 mM MgCl2, 50 pmol of each of the two primers and 1 U Taq DNA polymerase (Pharmacia Biotech, San Francisco, CA). After a denaturation step of 95°C for 4 min, amplification was carried out for 30 cycles of 95°C for 1 min, 1 min at the specific annealing temperature (**Supplementary Table 1**) and 72°C for 2 min, followed by 72°C for 7 min. PCR products were separated by agarose gel electrophoresis and either cloned in *Escherichia coli* or directly sequenced (Eurofins Genomics, Heidelberg, Germany). The nucleotide and deduced protein sequences from WT and *pat* line were compared between them and with those in the reference tomato sequence (SGN). A CAPS marker based on the G1747A transition found on the coding sequence of *Solyc03g120910* was developed by amplification with primers HD9 and HD10 and cutting with *Bfa*I (**Supplementary Table 1**).

### 2.3 *In silico* expression, protein structure prediction, and phylogenesis

Information about the expression of class III homeodomain leucine zipper (HD-Zip III) genes in tomato was obtained from TomExpress (http://tomexpress.toulouse.inra.fr/, accessed on 9 October 2023). To predict the effect of amino acid changes, the Sorting Intolerant from Tolerant (SIFT) software was used (http://sift.jcvi.org/). Sequence logos were created with WebLOGO (http://weblogo.berkeley.edu/logo.cgi), whereas I-TASSER was adopted to generate the predicted molecular models of WT and mutant proteins (http://zhang.bioinformatics.ku.edu/ITASSER/). Finally, the candidate protein was scanned using the Eukaryotic Linear Motif server (http://elm.eu.org/) searching for conserved sites.

HD-Zip III gene family members in tomato were identified by BLASTP at SGN using the *Arabidopsis* HD-Zip III protein sequences as queries: ATHB8 (*At4g32880*), ATHB9/PHV (*At1g30490*), ATHB14/PHB (*At2g34710*), ATHB15/CNA/ICU4 (*At1g52150*), and IFL1/REV (*At5g60690*). The *Arabidopsis* proteins and the six identified tomato orthologs were aligned by Geneious 5.6.3 (http://www.geneious.com/), and a phylogenetic tree was generated using Jukes-Cantor and neighbor-joining as genetic distance and tree building method, respectively. The phylogenetic analysis was performed with 1,000 bootstrap replicates. The protein sequences of AtHB15, SlHB15, and SlHB15-like were aligned using the ClustalW Multiple Alignment module implemented in BioEdit 7.2.5 (https://bioedit.software.informer.com/).

### 2.4 Gene expression analyses

Total RNA was isolated from 100 mg of whole WT and *pat* floral buds sampled 6 days before anthesis (referred to as −6 days post-anthesis, DPA) and ovaries at −4, −2, +2 DPA with TRIzol (Invitrogen, Carlsbad, CA, USA) following the manufacturer’s instructions. After extraction, 5 µg of RNA was used to synthesize cDNA by 1 U of Moloney murine leukaemia virus reverse transcriptase (Invitrogen) and a 3′-oligo(dT) primer in a final volume of 20 µl. quantitative real-time polymerase chain reaction (qRT-PCR) was performed using the Bio-Rad CFX96 Manager system (Bio-Rad, Hercules, CA, USA) with primers qHB15-1 and qHB15-2 (**Supplementary Table 1**), using three biological replicates. Amplification experiments were performed in a total volume of 15 µl, containing 1.75 µl of fourfold diluted cDNA, 1 X SSO ADV UNIVERSAL SYBR GREEN mix (Bio-Rad) and 300 nM of each primer. The amplification program included a denaturation step of 95°C for 30 s, followed by 40 cycles at 95°C for 5 s and 58°C for 30 s. To evaluate the gene expression level, results were normalized using the housekeeping clathrin adaptor complex subunit gene (CAC, *Solyc08g006960*). Gene expression was calculated according to the 2^-ΔCt^ formula.

For *in-situ* hybridization experiments, flower buds at −10 DPA, and excised pistils at −6 and −4 DPA from the WT and the *pat* mutant were fixed in FAA (ethanol, formaldehyde, and acetic acid) over night and then embedded in paraffin. Digoxigenin (DIG)-labeled RNA probes for detection and hybridization of *SlHB15* were prepared as previously described (Mizzotti et al., 2017). Eight μm-thick sections were hybridized with a DIG-labeled *SlHB15* antisense or sense probe, amplified using primers reported in **Supplementary Table 1**. Samples were observed using a Zeiss Axiophot D1 microscope (Zeiss, Oberkochen, Germany) equipped with differential interface contrast optics. Images were recorded with an Axiocam MRc5 camera using the Axiovision program (version 4.1).

### 2.5 Functional characterization by complementation and cosuppression

For the complementation and OE assay, the full-length *SlHB15* CDS was amplified from the cDNA of WT ovaries at the opening flower stage (−1 DPA). cDNA preparation was as described before. At both sides of the CDS, a cloning site was introduced using primers HD11 and HD12 (**Supplementary Table 1**). The PCR product was cloned in pGEM-T (Promega, Madison, WI, USA) and used to transform *E. coli* DH5α competent cells. Positive plasmids were cut to recover the full-length *SlHB15* CDS, which was introduced into the pBI121 vector under the control of the *CaMV35S* promoter and the *nopaline synthase* terminator.

The construct was sequence verified and used for *Agrobacterium tumefaciens* strain EHA105 transformation. Cotyledons of the WT (for OE/cosuppression) and of the *pat* mutant line (for complementation) were used for plant transformation following Gianoglio et al. (2022). Rooted primary transformants were checked for the presence of the transgene by PCR for the *SlHB15* cDNA and for the kanamycin resistance (*NptII*) gene (**Supplementary Table 1**). All mutant plants transformed for complementation were also checked to carry the *pat* allele using the CAPS marker described before.

### 2.6 Functional characterization by CRISPR/Cas9 gene editing

A single-guide RNA (sgRNA18) was designed to target *SlHB15* using CRISPR-P (http://cbi.hzau.edu.cn/crispr/). The GB CRISPR assembler (http://goldenbraid.com) was used for domestication of sgRNA18 and generation of the final gene-editing cassette. The domesticated 20 bp double-strand sequence homologous to sgRNA18 was generated by mixing 5 μl of 1 μM domesticated forward (CRI-HB15-1) and reverse (CRI-HB15-2) oligos (**Supplementary Table 1**) and letting them anneal for 30 min at room temperature. A transcriptional unit (CRISPR_TU) was generated by combining the double strand sequence, the Arabidopsis U6-26 promoter (GB1204) and the sgRNA (crRNA + tracrRNA; GB0645). CRISPR_TU was combined with the TU coding for the human codon optimized *hCas9* gene (GB0639) and with the TU for kanamycin resistance (GB0226). All construct assemblies were confirmed by restriction analysis and CRISPR_TU was checked by sequencing. The final construct (NptII:CRISPR-TU:hCas9) was inserted into *A. tumefaciens* strain LBA4404 to transform WT cotyledons as previously described.

To detect mutations in the gRNA18-Cas9 targeted *SlHB15* locus, genomic DNA was isolated from shoots of 20 out of 54 T_0_ regenerated plants showing alterations of the reproductive organs, plus one plant not showing such phenotypes. A T7E1 assay (Mashal et al., 1995) was performed on PCR products obtained with the CRI-HB15-3 and CRI-HB15-4 primers (**Supplementary Table 1**). To confirm CRISPR-Cas9-induced editing in the target region, amplicons showing a cleaved profile were Sanger-sequenced. TIDE (https://tide.nki.nl) was used for the detection of the predominant types of lesions in the DNA target sequence.

Potential off-target sites were identified by CRISPR-P. The four sequences with the highest risk of off-target effects, residing on the coding sequence of *Solyc04g074040*, *Solyc12g044410*, *Solyc03g124010*, and *Solyc08g066500* were PCR-amplified from a T_1_ plant for each of two lines selected for further studies using specific primer pairs (**Supplementary Table 1**). Amplicons were analyzed by TIDE.

### 2.7 Phenotypic and histological characterization

Reproductive aspects of the *pat* syndrome were analyzed in different phases and materials; only the number of plants and specimens varied, based on the number of available samples. Sample numbers are reported in the respective tables and figures.

Flowering time was recorded on a single plant basis as the day of opening of the first flower in the first truss and expressed as DPG. The percentage of aberrant stamens was estimated by dissecting at least three flowers per plant and counting the number of stamens having normal morphology or presenting aberrations typical of the *pat* mutant (shortness and/or carpelloidy). At anthesis, ovaries were dissected from at least three flowers per plant and weighted. Aberrant ovules were then counted under a stereomicroscope after dissecting pieces of placenta with a razor blade (Mazzucato et al., 1999); ovule aberrancy was expressed as the percentage of abnormally developed ovules over a total of 40–60 observed per ovary. To assess parthenocarpic capacities, flowers were emasculated before anthesis (−2 DPA), tagged, and left unpollinated. Ten days post-emasculation (DPE), ovaries/fruitlets were dissected and weighted.

Fruit weight was estimated at maturity on a minimum of ten fruits per plant and five plants per line. To estimate the seed set, a minimum of ten open-pollinated fruits were collected at maturity from five to 10 plants; seeds were extracted, dried, counted, and finally reported on a single fruit basis. On WT and *SlHB15*-edited plants, the number of ripe and unripe fruits was counted and used to estimate respectively the actual and potential yield per plant by referring to the mean fruit weight. In addition, on a representative number of fruits, locule number was counted and the fruit puffiness scored (1, absent; 2, weak; 3, strong). On a single plant basis, the soluble solids content (SSC) was measured with a digital MA871 refractometer (Milwaukee Instruments, Inc., NC, United States) and expressed in Brix degrees (°B). The SSC production per plant was calculated using SSC and the actual plant yield.

Vasculature development was investigated in WT and *pat* 30-day-old plantlets using six individuals per genotype cultured in a growth chamber at 24 ± 2°C under 16/8h light/dark photoperiod. Handmade cross sections of the hypocotyl and the epicotyl, about 1.5 cm below and above the cotyledon insertion, respectively, were stained in a phloroglucinol-satured solution 20% HCl in the dark for 15 min (Jensen, 1962) and immediately photographed under the stereomicroscope. To detect vasculature development in fruitlets/fruits, a WT and a *pat* line transformed with an IAA-reporter construct (Mazzucato et al., 2006) were used. Fruitlet or fruit specimens were dissected at 6 and 11 DPA and at the mature green stage, hand-sectioned, stained for the GUS assay, and photographed with a stereomicroscope (Mazzucato et al., 2006).

To observe the phenotype of *HB15* alleles, 20 *Arabidopsis* plants each for the *cna-1* and *icu4-1* mutants and their respective WT lines (Col-0 and En-2) were first investigated for the presence of cotyledon alterations by stereomicroscopy on 5-DPG seedlings. Plantlet phenotype was documented at 15 DPG and the number of rosette leaves recorded at 25 DPG. Twelve plants per genotype were used to estimate the flowering time, recorded as DPG to the emergence of the first inflorescence. In the same plants, flower organ, silique, and seed development were observed on dissected specimens by stereomicroscopy. To study parthenocarpic attitudes, 12 flowers for each genotype were emasculated before anthesis, tagged, and left unpollinated. Ten-DPE ovaries/fruitlets were dissected from emasculated flowers and weighted.

### 2.8 Statistical analyses

The map position of markers located in the target region was refined by testing the recombination rate in the mapping populations for the *pat* gene. JoinMap 4.1 (https://joinmap.software.informer.com/4.1/) was used to perform the linkage analysis and to integrate the map distances of the two mapping populations: an logarithm of the odds (LOD) score of 3.0 or above was specified. The Kosambi mapping function was used to convert recombination frequencies into map distances.

Pairwise mean comparison was performed using Student’s *t* test. Where a multiple comparison was expected, data analysis was conducted by general linear model (GLM) and tested for significance of differences among means at the 5% level (Duncan test). All statistical analyses were performed with the SAS software (https://www.sas.com/en_us/software/on-demand-for-academics.html).

## 3 Results

### 3.1 Positional cloning of the *pat* mutation

**Figure 1:**
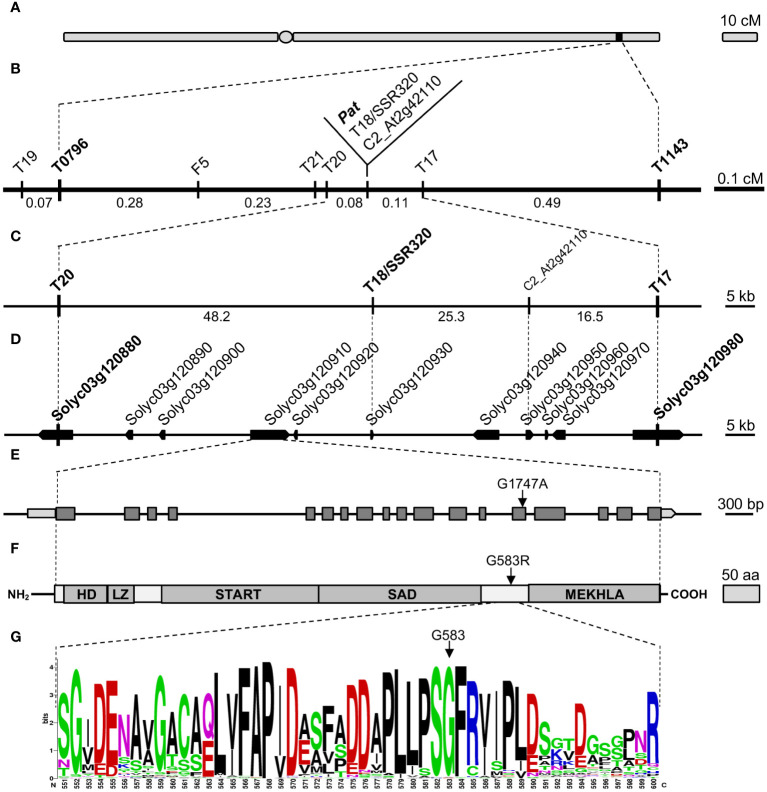
Fine mapping and candidate gene identification for the *Pat* locus. **(A)** Schematic representation of the tomato Chr3 with the position of the *Pat* locus, **(B)** target window narrowed to 0.19 cM between markers T20 and T17, **(C)** physical map of the T20–T17 region according to the published genome (ITAG 4.0), **(D)** annotation of the T20–T17 region according to the current genome annotation, **(E)** *SlHB15* gene model (*Solyc03g120910.4.1*), light gray boxes indicate 5′ and 3′ UTRs, dark gray boxes and black horizontal lines represent exons and introns, respectively. The arrow points the G1747A transition found in the 14th exon of the *SlHB15^pat^* allele. **(F)** Schematic representation of the SlHB15 protein; HD, LZ, START, SAD, and MEKHLA represent the five HD-Zip III characteristic domains, the arrow points the G583R amino acid change occurring in the SlHB15*^pat^* protein, **(G)** WebLOGO of a 50 amino acid portion between the SAD and MEKHLA domains of the SlHB15^WT^ protein showing the high degree of conservation of G583 (arrow).

To refine the 1.2-cM genetic window spanning the *pat* locus (Beraldi et al., 2004; **Figure 1A**), novel markers were developed inside the target region (**Figure 1B**, **Supplementary Data**). Finally, T18/SSR320 and C2_At2g42110 co-segregated with *pat* in the refined genetic region of 0.19 cM, flanked by markers T20 and T17 (**Figure 1B**).

Because markers T20 and T17 corresponded to *Solyc03g120880* and *Solyc03g120980*, respectively, the *pat* region spanned about 90 Kbp (**Figure 1C**) and contained nine annotated genes (**Figure 1D**). Of these, three encoded proteins of unknown function (*Solyc03g120920*, *Solyc03g120940*, and *Solyc03g120950*), two encoded transcription factors (TFs; *Solyc03g120890*, a GATA protein, and *Solyc03g120910*, a HD-Zip III protein), two encoded small proteins involved in the host-parasite (*Solyc03g120930, Avr9/Cf9*) and pollen-pistil (*Solyc03g120960, STIG1*) interaction, respectively, one encoded a SEC13-like transport protein (*Solyc03g120900*) and one a GA 2-beta-dioxygenase (*Solyc03g120970*, **Supplementary Table 2**).

cDNA and genomic DNA sequences were obtained for the nine candidate genes in the WT and in the *pat* mutant genotypes. A single SNP was found in the coding sequence of *Solyc03g120910*, corresponding to a G to A base change in exon 14 (**Figure 1E**) causing a glycine to arginine substitution (G583R) in the protein sequence (**Figure 1F**). The mutation, assayed as a CAPS marker (not shown), co-segregated with the *pat* phenotype when tested on recombinant plants within the target window. Individuals heterozygous for the mutation showed an intermediate phenotype for *pat* syndrome traits, such as aberrant stamens, fruit weight and number of seeds per fruit (**Supplementary Figure 1**). Thus, mapping, phenotypic and sequence data suggested the G1747A mutation in *Solyc03g120910* as the putative basis of the *pat* phenotype.

*Solyc03g120910* encodes a HD-Zip III homeobox protein with similarity to *HB15* in *Arabidopsis* [also known as *CORONA* (*CNA*) or *INCURVATA4* (*ICU4*)]; the gene was consequently referred to as *SlHB15*. In the *pat*-mutant protein, the G583R substitution was located between the SAD and MEKHLA domains typical of HD Zip III proteins (**Figure 1F**) and involved a highly conserved sequence of four amino acids (PSGF; **Figure 1G**, **Supplementary Figure 2**). This substitution was predicted to be deleterious by SIFT analysis (not shown), and strong structural differences in the mutant protein were foreseen based on 3D models (**Supplementary Table 3**; **Supplementary Figure 3A**).

**Figure 2:**
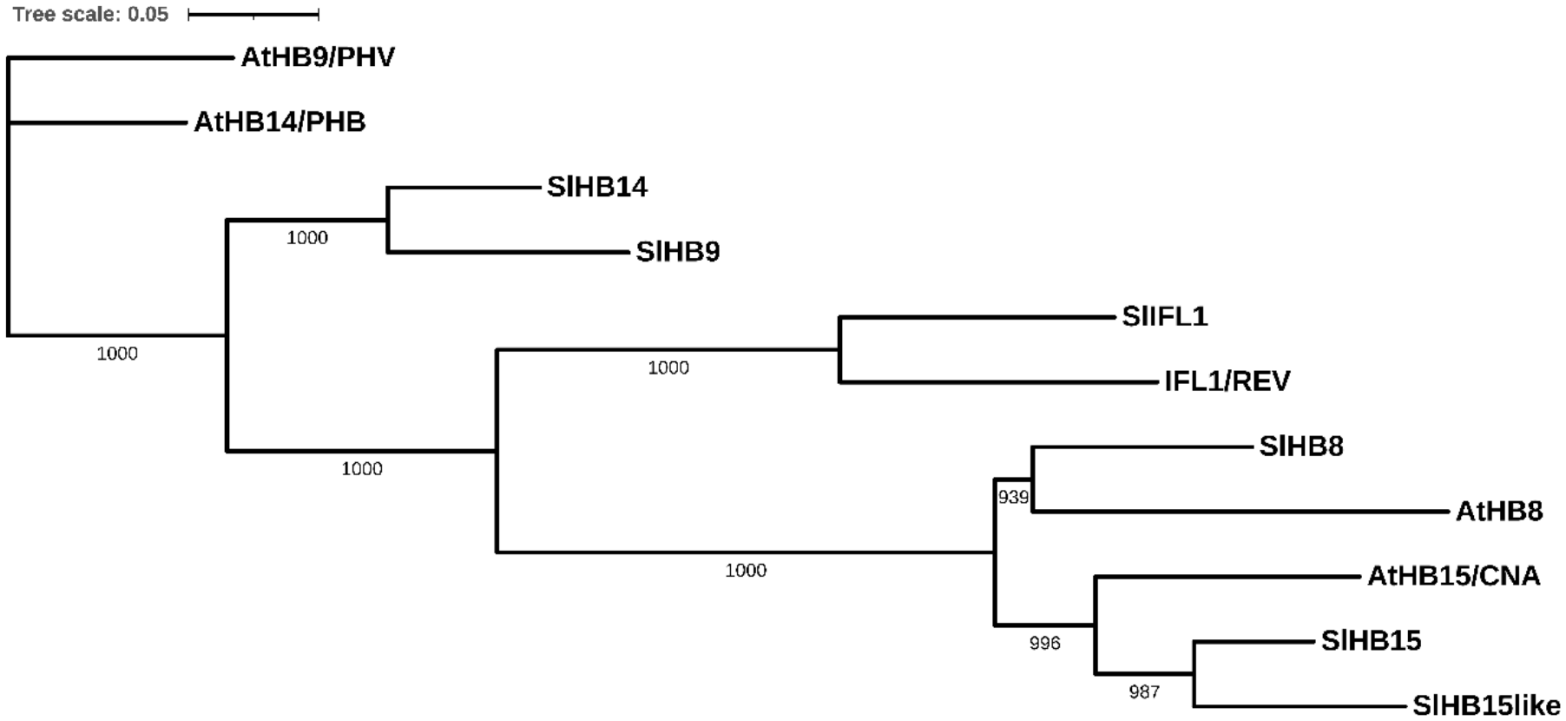
Neighbor-joining tree based on the full-length protein alignment of tomato and arabidopsis HD-Zip III proteins. The tree shows percentage bootstrap support at each node (*n* = 1,000) for the five Arabidopsis proteins: PHAVOLUTA (PHV)/AtHB9, PHABULOSA (PHB)/AtHB14, REVOLUTA (REV)/INTERFASCICULAR FIBERLESS1 (IFL1), AtHB8, and CORONA (CAN)/AtHB15/ICU4, and for the six tomato orthologs, respectively: SlHB9, SlHB14, SlIFL1, SlHB8, SlHB15, and SlHB15-like. Scale bar = number of amino acid substitutions per site.

In *Arabidopsis*, the HD-Zip III subfamily includes five members, *PHAVOLUTA* (*PHV*)/*AtHB9*, *PHABULOSA* (*PHB*)/*AtHB14*, *REVOLUTA* (*REV*)/*INTERFASCICULAR FIBERLESS1* (*IFL1*), *AtHB8*, and *AtHB15*/*CNA*/*ICU4*; the tomato genome includes all ortholog members, respectively, *SlHB9*, *SlHB14*, *SlIFL1*, SlHB8, and *SlHB15*. In addition, *SlHB15* presented a paralog gene on Chr12 (*Solyc12g044410*) that is not found in *Arabidopsis* (hereafter referred to as *SlHB15-like* (**Figure 2**; **Supplementary Figure 3B**).

### 3.2 *SlHB15* is differentially expressed in reproductive tissues of the WT and the *pat* mutant

**Figure 3:**
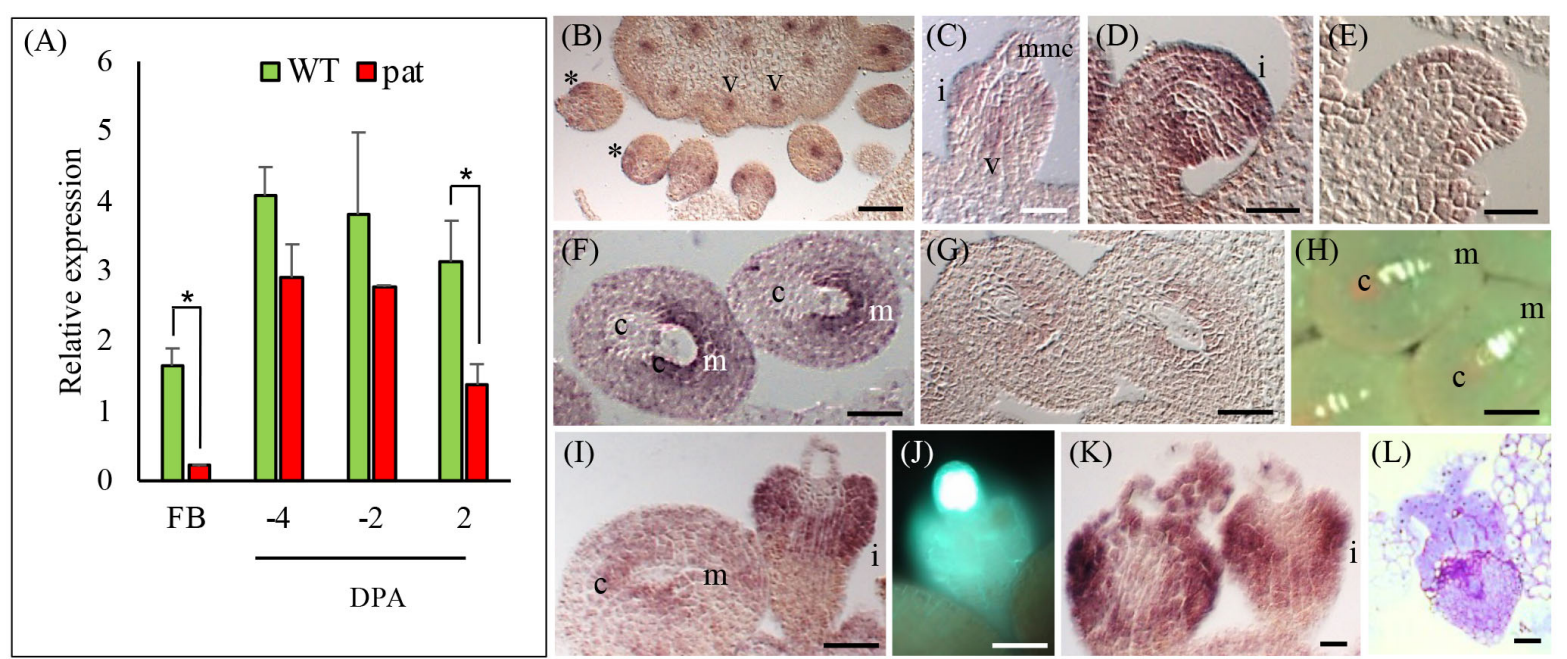
*SlHB15* expression profile in the WT and the *pat* mutant and *in-situ* detection of its transcripts. **(A)** RT-PCR expression data of *SlHB15* in the WT (green bars) and in the *pat* mutant (red bars) in flower buds at −6 days post-anthesis (DPA, FB) and in ovaries at −4, −2, and 2 DPA. Values are means ± SE; *n* = 4. * indicates significant difference between genotypes within tissue for *p* ≤ 0.05. **(B)** Section of WT pistil hybridized with a *SlHB15* antisense probe at Stage −10 DPA; expression is visible in the emerging ovule integument (*asterisk*) and in the vascular tissues of the pistil (V). **(C)** Closer view of a developing WT ovule at −10 DPA with *SlHB15* messengers present in the developing integument, as well as in the funicular vasculature. **(D)** *SlHB15* expression in ovule integument at −6 DPA mainly in the region surrounding the micropyle. **(E)** Same as **(D)** hybridized with the sense probe. **(F)** Hybridization signal in WT ovules at −4 DPA; **(G)** Same as **(D)** hybridized with the sense probe. **(H)** Callose staining highlighting the female tetrads at −6 DPA. **(I)** *pat*-mutant ovule at −6 DPA with *SlHB15* accumulation less evident in normal-like ovules, but strong in the integument of the aberrant ovules. **(J)** Aberrant ovule with callose staining at the megaspore mother cell site. **(K)** Gene expression still strongly detected in the aberrant ovules at −4 DPA, that seldom **(L)** presented proliferation of the nucellar tissue. v, vasculature; i, integument; mmc, megaspore mother cell; c, chalaza; m, micropyle. Scale bar is 25 µm in **(B–E)** and 50 µm in **(F–L)**.

According to published data (http://tomexpress.toulouse.inra.fr/, accessed on 9 October 2023), *SlHB14*, *SlHB15, SlHB15-like* and *SlIFL1* showed remarkably similar expression patterns in vegetative and flower meristems and in whole flowers of WT (non parthenocarpic) tomato (**Supplementary Figure 4A**). At stages spanning fruit set (0–5 DPA), *SlHB15* and *SlIFL1* showed the highest expression in ovules and in the pericarp; *SlHB15* showed an evident developmentally regulated expression in ovules (**Supplementary Figures 4B, C**). The expression pattern of *SlHB15* in floral organs of the WT and the *pat* mutant were quantified by qRT-PCR. In flower buds, the *pat* mutant had lower *SlHB15* expression values; in ovaries, *SlHB15* expression was lower in the mutant, being significant at 2 DPA (**Figure 3A**). Expression of *SlHB15-like* in ovaries paralleled that of *SlHB15* but was lower, without significant differences between the WT and the mutant (**Supplementary Figure 4D**). The expression of the two paralogs retrieved from published microarray results (Ruiu et al., 2015) closely paralleled that estimated by qRT-PCR (**Supplementary Figures 4E, F**).

To study *in situ*, the expression of *SlHB15*, sections of WT and *pat*-mutant ovaries were hybridized with a *SlHB15* antisense probe. In WT ovaries at a pre-meiotic stage of about −10 DPA, *SlHB15* was expressed in the emerging ovule integument and in the vascular tissue of the placenta (**Figure 3B**). In ovules, *SlHB15* expression defined a ring around the nucellus (asterisk) in the integument primordia, as well as in the funicular vasculature (**Figure 3C**) and persisted in the integument when it grows surrounding the nucellus (**Figure 2D**). At a later stage, when tetrads have formed (−6 DPA), *SlHB15* maintained its expression in the integument with transcripts accumulating mainly in the micropylar region (**Figures 3F, H**). In the *pat* mutant, accumulation was less evident in WT-looking ovules at the same stage, while it was still strongly detected in the aberrant ones (**Figure 3I**), where meiosis is arrested and the female meiocyte remains exposed with strong callose deposition (**Figure 3J**). Strong expression persisted at later stages in club-shaped *pat* aberrant ovules (**Figures 3K, L**). No signal was detected in negative controls with the sense probe (**Figures 3E, G**).

*Arabidopsis* HD-Zip III proteins are involved in vasculature development (Prigge et al., 2005); therefore, we analyzed the vascular phenotype in WT and *pat*-mutant tomato seedlings. Compared to the WT, hypocotyl cross sections of *pat* plantlets showed a disorganized vasculature (**Supplementary Figures 5A, B**), although the symmetry of the vascular bundles appeared to be conserved. Vascular bundles disposition was different between hypocotyl and epicotyl, but, compared to WT plantlets, the disorganized vasculature scored in the *pat* hypocotyl was reflected at the epicotyl level (**Supplementary Figures 5C, D**). Vasculature development was monitored in the developing fruit by using an auxin reporter gene; in the mutant, fruitlet vascular bundles were more developed than in the WT at parallel developmental stages (**Supplementary Figures 5E–J**).

### 3.3 Functional analysis confirmed *SlHB15* as the causative gene of the *pat* syndrome

**Figure 4:**
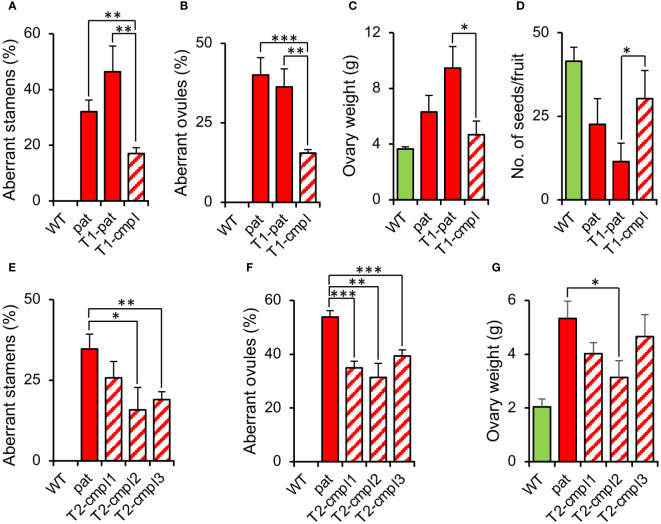
Characterization of plants complementing the *pat* mutation. Percentage of **(A)** aberrant stamens and **(B)** aberrant ovules, **(C)** ovary weight at anthesis, and **(D)** mean number of seeds per fruit in T_1_ plants transformed with the *35S:SlHB15^WT^* construct non-showing (T1-pat) or showing (T1-cmpl) signs of complementation in comparison with the WT and *pat* mutant line. Percentage of **(E)** aberrant stamens and **(F)** aberrant ovules, and **(G)** ovary weight at anthesis in three T2 progenies showing complementation in comparison with the WT and *pat* mutant line. Data are means obtained by at least ten flowers or plants. *, **, and *** indicate means significantly different from the *pat* mutant or from transformed plants non-showing complementation (T1-pat) after Student’s t-test.

To confirm that the *SlHB15* mutation was responsible for the *pat* phenotype, we attempted to complement the mutant phenotype by expressing *SlHB15* under a constitutive promoter. T_1_ progeny individuals showed a wide variety of phenotypes, ranging from plants severely presenting the complete *pat* syndrome to plants showing attenuated *pat* phenotypes. Considering the decrease of the frequency of aberrant ovules as a criterium, eight T_1_ plants were considered partially complemented (T_1_-cmpl) and eight were classified as non-complemented (T_1_-pat). Compared to T_1_-pat, T_1_-cmpl plants showed on average a significantly reduced frequency of aberrant stamen and ovules (**Figures 4A, B**), a reduced ovary weight at anthesis (**Figure 4C**) and an increased number of seeds per fruit (**Figure 4D**). T_2_ progenies confirmed the mitigation of *pat* traits, such as the frequency of aberrant stamens and ovules and the increased ovary weight at anthesis (**Figures 4E–G**).

**Figure 5:**
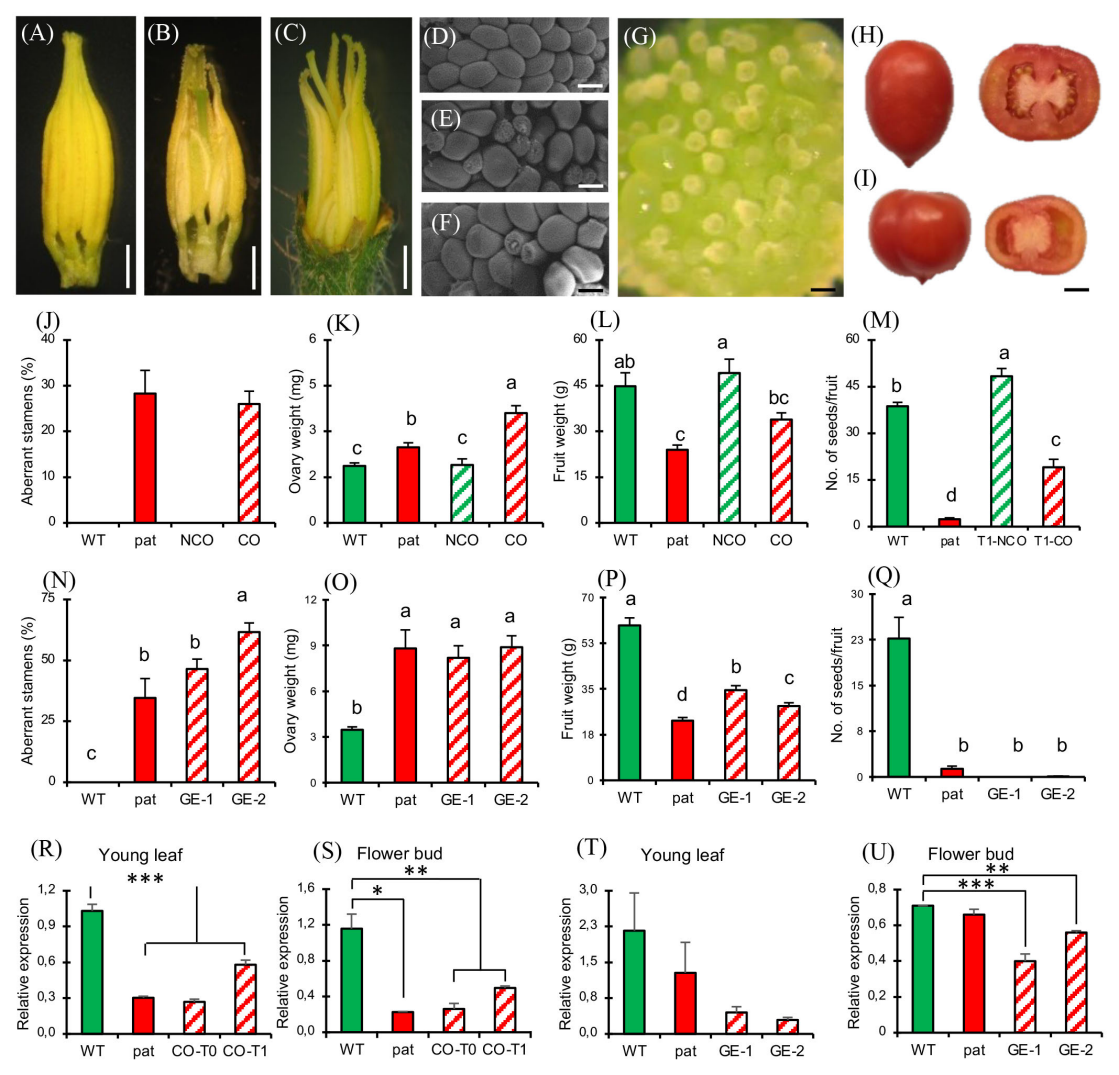
Phenotypic and molecular characterization of plants silenced for *SlHB15* by cosuppression and CRISPR. Dissected staminal cones from **(A)** WT, **(B)** cosuppressed T_0_, and **(C)** CRISPR knock out plants. Dissected portion of the placenta and ovule phenotype in **(D)** WT, **(E)** *pat* mutant, **(F)** cosuppressed, and **(G)** edited plants. Representative entire and transversally sectioned fruits of a **(H)** WT and of an **(I)** edited plant. **(J)** Percentage of aberrant stamens, **(K)** ovary weight at anthesis, **(L)** ripe fruit weight, and **(M)** number of seeds per fruit in WT and *pat* plants in comparison with WT plants transformed with the *35S:SlHB15* construct without (NCO) or with (CO) signs of cosuppression. Data are means ± SE, *n* = 96, 32, 32, 32 for **(J-M)**, respectively. Means indicated by the same lowercase letter are not significantly different for *p* ≤ 0.05 after Duncan multiple range test. **(N)** Percentage of aberrant stamens, **(O)** ovary weight at anthesis, **(P)** ripe fruit weight and **(Q)** number of seeds per fruit in WT and *pat* plants in comparison with two independent lines knocked out for *SlHB15* by gene editing (GE-1, GE-2). Data are shown as means ± SE, *n* = 96, 32, 32, 32 for **(N-Q)**, respectively. Means indicated by the same lowercase letter are not significantly different for *p* ≤ 0.05 after Duncan multiple range test. *SlHB15* expression in **(R)** young leaves and in **(S)** flower buds at −6 days post-anthesis (DPA) in WT and *pat* plants in comparison with cosuppressed plants in the T_0_ (CO-T0) and in the T_1_ (CO-T1) generation. *SlHB15* expression in **(T)** young leaves and in **(U)** flower buds at −6 DPA in WT and *pat* plants in comparison with two independent *SlHB15^CRISPR^* lines (GE-1, GE-2). Data are shown as means ± SE, *n* = 3. *, **, and *** indicate significant difference from the WT for *p* ≤ 0.05, 0.01, and 0.001 after Student’s t-test. Bar is 2 mm in **(A–C)**, 100 µm in **(D–G)**, and 1 cm in **(H, I)**.

When WT plants were transformed with the constitutive *35S:SlHB15^WT^* construct, 36 regenerants were indistinguishable from untransformed WTs for both vegetative and reproductive aspects (not shown). However, two T_0_ individuals (referred to as CO plants) showed *pat*-like reproductive defects, with aberrant stamens (**Figures 5B, J**) and ovules (**Figure 5F**), and bigger ovaries at anthesis (**Figure 5K**). To test the stability of the *pat* phenotypes in CO plants, a T_1_ progeny was grown and analyzed. Eight out of 28 T_1_ seedlings were nullisegregant, whereas the others carried the transgene (Mendelian segregation 3:1, χ^2^ 0.19, *p* > 0.05). Among transgenics, several polycotyledonary seedlings were observed (not shown), at a frequency even higher than that found in the *pat* mutant (22%; Olimpieri et al., 2007). Nullisegregants showed a WT floral phenotype, while 17 out of 20 T_1_ transgenic plants showed alterations of stamens, ovules, and ovaries comparable to the T_0_ parent plant and to the *pat* mutant. In addition, such T_1_ individuals paralleled the *pat* mutant having smaller fruits (**Figure 5L**) and a lower number of seeds (**Figure 5M**) compared with the respective WTs. However, these plants neither produced completely seedless fruits nor were able to develop fruits from emasculated flowers. The expression of *SlHB15* in T_0_ and T_1_ CO plants in young leaves and flower buds was strongly downregulated, thus paralleling the repressed expression in the mutant (**Figures 5R, S**) and supporting that some aspects of the *pat*-mutant phenotype are due to *SlHB15* cosuppression.

To provide further evidence that the loss of function of *Solyc03g120910* underlies all aspects of the *pat* phenotype, we generated CRISPR/Cas9 knockouts targeting the third exon of the gene. Of 42 transgene-positive regenerants, 21 were sequenced, revealing a variety of genetic lesions (**Supplementary Table 4**) and phenotypes; two lines showing a severe phenotype already in T_0_ (hereafter referred to as GE-1 and GE-2) were further propagated and studied. GE-1 presented a 5-bp deletion, whereas GE-2 showed a biallelic mutation including an A insertion and a 7-bp deletion in the coding region of *Solyc03g120910* exon 3 (**Supplementary Figure 6B**).

In both cases, mutations caused a frame shift and a premature stop codon in the transcript. The four predicted off-target sites, residing in the coding sequence of *Solyc04g074040*, *Solyc12g044410*, *Solyc03g124010*, and *Solyc08g066500*, did not show any detectable mutation (not shown). The two edited lines strongly recapitulated the *pat*-like floral phenotypes, such as aberrant stamens (**Figures 5C, N**) and aberrant ovules (reaching almost 100%, **Figure 5G**). As in *pat*, the ovary weight at anthesis in the edited lines was significantly higher than in the WT (**Figure 5O**). Compared to the WT fully seeded fruits, edited fruits were smaller (with less seeds; **Figures 5I, P**), as in the *pat* mutant (**Figure 5Q**). In edited lines, *SlHB15* expression was downregulated compared to the WT (**Figures 5T, U**), thus paralleling the decrease in expression detected in cosuppressed plants.

**Figure 6:**
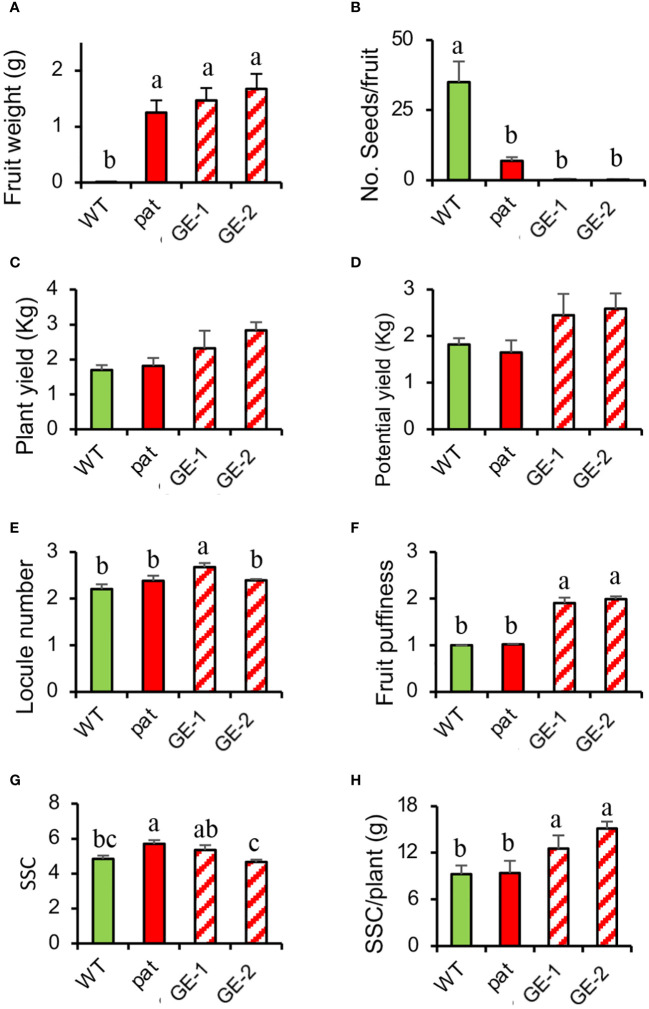
Fruit traits and yield estimation in *SlHB15^CRISPR^* plants compared with the WT and the *pat* mutant. **(A)** Fruit weight 13 days after emasculation, **(B)** mean number of seeds per fruit, **(C)** actual and **(D)** potential yield, **(E)** locule number, **(F)** fruit puffiness, **(G)** soluble solid content (SSC), and **(H)** soluble solid production per plant in WT and *pat* plants in comparison with two independent knock-out *SlHB15^CRISPR^* lines (GE-1 and GE-2). Data are shown as means ± SE, *n* = 24 for **(A, B, E, F)** and 4 for **(C, D, G, H)**. Means indicated by the same lowercase letter are not significantly different for *p* ≤ 0.05 after Duncan multiple range test.

In T_1_, the increased number of edited plants allowed a wider analysis on fruits traits. An emasculation experiment confirmed the strong parthenocarpic capacity of GE-1 and GE-2 as the mean weight of fruitlets developed from emasculated, not pollinated flowers indicated considerable ovary development, whereas the WT could not set any fruit in such conditions (**Figure 6A**). Such fruits ripened as those from open-pollinated ovaries but contained almost no seed (**Figure 6B**). When left to open pollination, *SlHB15^CRISPR^* plants showed a yield higher than both the WT and the *pat* mutant (**Figures 6C, D**), although it did not reach the statistical threshold. Whereas *SlHB15^CRISPR^* fruits showed a similar number of locules as the WT and the mutant (**Figure 6E**), they presented a higher puffiness occurrence (**Figure 6F**). Finally, the SSC content did not differentiate the four lines (**Figure 6G**), but when it was related to the potential yield, the SSC production of *SlHB15^CRISPR^* lines overtook that of the WT and of the *pat* mutant by 2–4 g/plant (**Figure 6H**).

### 3.4 *Arabidopsis HB15* mutants partially recapitulate *pat* alterations

**Figure 7:**
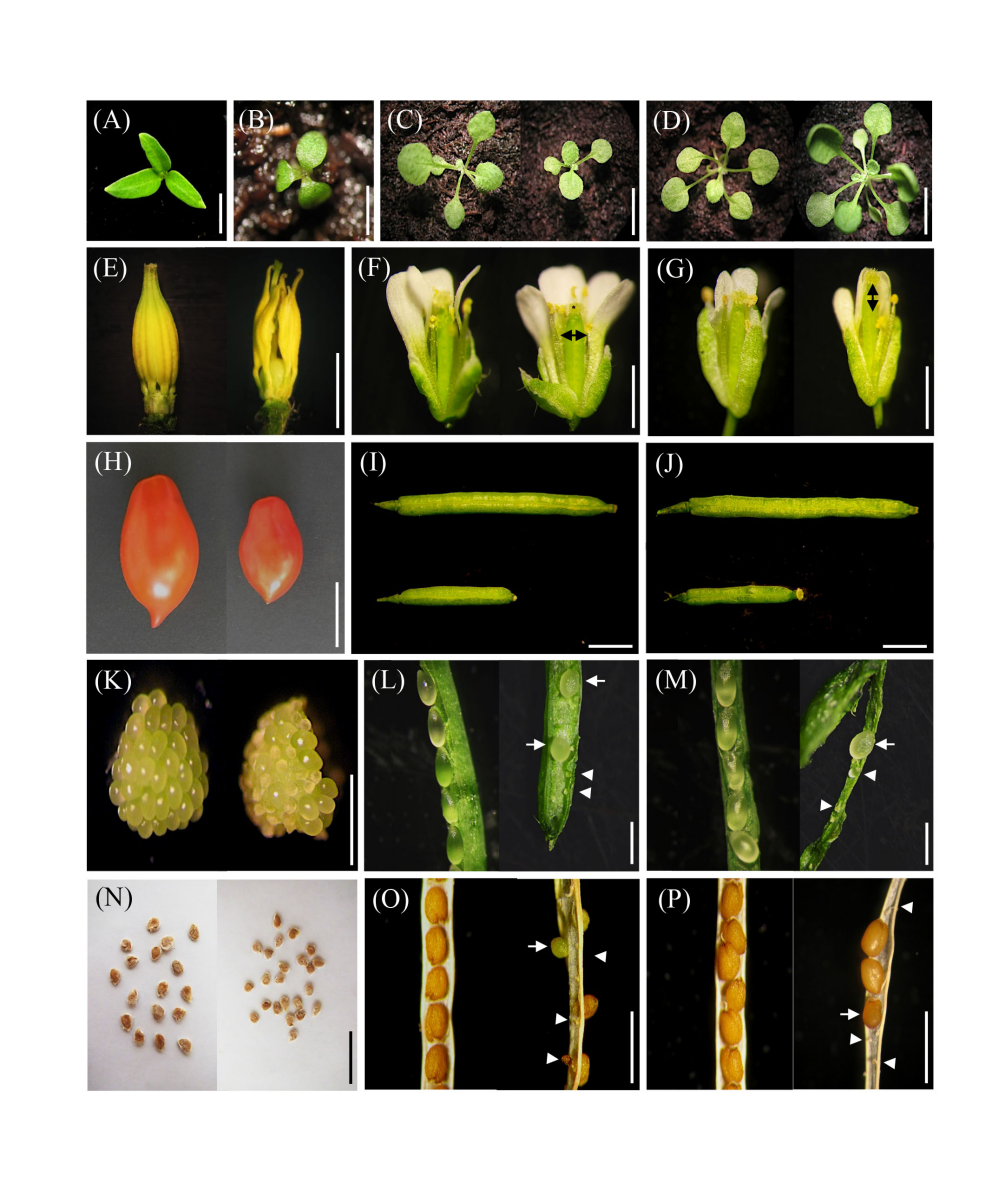
Vegetative and reproductive phenotypes of the *pat* mutant in parallel with those of *cna-1* and *icu4-1 Arabidopsis HB15* mutants. **(A)** tomato and **(B)** *Arabidopsis* tricot seedlings in the *pat* and *icu4-1* mutants, respectively. Plantlet phenotype of **(C)** Col-0 and *cna-1* at 15 days after germination (DAG; in every panel, the WT is on the left and the mutant on the right). Plantlet phenotype of **(D)** En-2 and *icu4-1* at 15 DAG. Staminal cone at anthesis in **(E)** WT and *pat* flowers; the *pat* staminal cone at anthesis shows the bigger size of the *pat* ovary compared to WT. **(F)** Col-0 and *cna-1* flower and pistil at anthesis; in the mutant a thicker pistil is evident (*horizontal double arrowhead*). **(G)** En-2 and *icu4-1* flower and pistil at anthesis; in the mutant a longer pistil is evident (*vertical double arrowhead*). Mature fruit phenotype of **(H)** WT and *pat* plants, of **(I)** Col-0 and *cna-1* mutant and of **(J)** En-2 and *icu4-1* mutant. Ovule phenotype in the **(K)** WT and *pat* ovary at anthesis; in the mutant, aberrant ovules show impaired growth of the integument. Ovule/seed phenotype at 7 days post-anthesis (DPA) in **(L)** the Col-0 and *cna-1* and in **(M)** the En-2 and *icu4-1* mutant silique; subnormal (*arrows*) and aberrant ovules (*arrowheads*) are evident in the mutants. Seed phenotype of **(N)** WT and partially seeded *pat* mutant fruits, and of **(O)** Col-0 and *cna-1* and **(P)** En-2 and *cu4-1* dry siliques; subnormal (*arrow*) and aborted (*arrowheads*) seeds in mutant siliques are evident. Scale bar is 2 cm in **(A)**; 1 cm in **(B–D)** and **(N)**; 5 mm in **(E)**; 1 mm in **(F, G, K)**, and **(O, P)**; 5 cm in **(H)**; 2 mm in **(I, J)**; and 0.5 mm in **(L, M)**.

The phylogenetic analysis indicated that *Solyc03g120910* represents the tomato ortholog of *HB15* in *Arabidopsis*. To evaluate whether *HB15* variants showed phenotypes resembling those found in *pat* in tomato, we characterized targeted vegetative and reproductive traits in *cna-1*, a loss-of-function mutant showing a A606V substitution in a conserved domain (Green et al., 2005) and *icu4-1*, a gain of function variant showing a point mutation affecting the microRNA complementarity site (Ochando et al., 2006). About 22% of the *pat* mutant seedlings showed defects in cotyledon number and/or morphology (Olimpieri et al., 2007; **Figure 7A**); a similar phenotype was found in about 10% of the *icu4-1* seedlings (**Figure 7B**), but no cotyledon alterations were observed in *cna-1*. *pat* mutant plants have been reported to be smaller than the WT at different time points (Mazzucato et al., 1999); compared to its WT counterpart, *cna-1* also showed a general reduction in plant size and in the number of rosette leaves (**Figure 7C**). Differently, *icu4-1*, compared to its WT reference, had the opposite effect (**Figure 7D**). When compared to Col-0 plants, *cna-1* showed an early flowering phenotype similarly to the tomato *pat* mutant, whereas *icu4-1* did not display any significant reduction of the flowering time (**Table 1**).

**Table 1:** Flowering time and ovary weight after emasculation in *pat* and in *Arabidopsis HB15* mutants.

Species	Genotype	Flowering time		Ovary weight (mg)/silique length (mm) at			
		DPG	*p*	0 DPE	*p*	10 DPE	*p*
Tomato	WT (Chico III)	51.8	**	3.6	***	–	***
	*pat*	48.5		8.3		741.7	
*Arabidopsis*	WT (Col-0)	26.8	***	2.0	ns	2.2	**
	*cna1*	21.7		1.9		4.0	
	WT (En-2)	22.5	ns	2.1	**	2.5	*
	*icu4.1*	21.2		2.6		5.2	

Flowering time expressed as days post-germination (DPGs) in WT and *pat* tomato plants, and in *Arabidopsis* Col-0 and *cna-1* loss-of-function mutant, and En-2 and *icu4-1* gain-of-function mutant. Ovary weight (mg) in WT and *pat* tomato plants at anthesis zero and ten days post-emasculation (DPE) and silique length (mm) in Col-0 and *cna-1* and En-2 and *icu4-1* at the same stage. Data are means of 12 plants or ovaries. *, **, and *** indicate significant differences between the WT and the mutant after Student’s t-test for *p* ≤ 0.05, 0.01, and 0.001, respectively.

ns, not significant.

Interestingly, pistils at anthesis of both *cna-1* and *icu4-1* appeared respectively thicker (**Figure 7F**) and longer (**Figure 7G**) than their WT counterparts. This is reminiscent to the larger size of the tomato *pat* ovary at the anthesis, indicating they have already started autonomous parthenocarpic growth (**Table 1** and **Figure 7E**). The parthenocarpic capacity of tomato ovaries to develop autonomously (about 50% emasculated flowers in *pat* set fruit) was paralleled also by *Arabidopsis* mutants where emasculated not-pollinated flowers developed in 10 DPE parthenocarpic siliques significantly longer than those produced by their respective WTs (**Table 1**). Similarly, to *pat* in tomato, that shows fruits significantly smaller than the WT (**Figures 4D**, **5L, P**, **7H**), parthenocarpic mature *cna-1* and *icu4-1* siliques were respectively about 40% and 30% shorter than those formed after self-pollination (**Figures 7I, J**). Ovular aberrations paralleling those showed by *pat* in tomato (**Figure 7K**) were also observed in both *cna-1* and *icu4-1* at 7 DPA, whereas the respective WTs had normal ovule development (**Figures 7L, M**). As a result of these aberrations, *cna-1* and *icu4-1* had a limited seed set and seeds were smaller than in the respective WTs (**Figures 7O, P**), as it happens in the *pat* tomato mutant (**Figure 7N**).

## 4 Discussion

### 4.1 Genetic mapping, molecular characterization, and *in-silico* analysis indicate *SlHB15* as the candidate for the *pat* mutation

Genetic and physical mapping, together with sequence and expression analyses, indicated *Solyc03g120910* as the gene candidate to underlie the *pat* mutation. Overall, the reported G1747A transition and the derived predictions supported the hypothesis that this variation caused the mutant phenotype through modification of the translated protein. The *Arabidopsis* ortholog of *Solyc03g120910*, *HB15*/*CNA*, has been involved in the regulation of critical aspects of plant development such as ovule polarity, apical and lateral meristem formation, and vascular development (Kim et al., 2005; Ochando et al., 2006; Kelley et al., 2009). The mutation underlying the *pat* phenotype involved a highly conserved residue, that was predicted to be important for the protein function.

*SlHB15* is expressed in flowers and fruits, suggesting a major role during tomato reproduction. *SlHB15* expression level was lower in *pat* mutant reproductive tissues compared to the WT. In tomato, a functional characterization of HD-Zip III genes has recently been described after the cloning of the *parthenocarpic fruit 1* (*pf1*) mutation (Clepet et al., 2021). Among the *SlHB15* EMS mutants described, the missense mutation *pf1-19* (T560I) was in the same region as *pat*; however, the residue affected in *pf1-19* was neither conserved nor the mutant showed a parthenocarpic phenotype (**Supplementary Figure 5** in Clepet et al., 2021).

For the *pat* mutation, SIFT prediction indicated the G583R substitution as non-tolerated. Accordingly, the prediction of the mutated protein structure indicated possible modifications altering domains important for its TF activity (HD, LZ, and START), that could affect its functionalities, such as the interaction with the DNA of target gene promoters and homo- or heterodimerization. Overall, such predictions suggested that functional modifications of the SlHB15^pat^ protein underlie the *pat* mutation.

### 4.2 *pat* phenotypes are recapitulated by plants silenced or knocked out for *SlHB15*

Complementation and knockout experiments supported the hypothesis that *pat* phenotype is due to the loss of SlHB15 function. *SlHB15* knock-out by genome editing yielded lines with a phenotype more severe than in the *pat* mutant. This may depend on the type and position of the mutation, that in the case of *pat* is a hypomorphic point mutation in the 14th exon and in the case of CRISPR lines was a frameshift/non-sense lesion in the third exon. We addressed the editing to this region of the gene, because it represented the best choice to avoid off-target effects. Overall, the edited plants recapitulated all the *pat* mutant phenotypes; in both T_0_ and T_1_, *SlHB15^CRISPR^* plants showed almost 100% ovule aberrancy with a consequent almost complete female sterility. Although such plants could hardly be useful in a seed-propagated crop, they may be a good approach to guarantee fruit production and complete seedlessness in vegetatively propagated crops, where this is an important quality aspect.

### 4.3 SlHB15 acts as a fruit set repressor and its disruption causes parthenocarpy in the *pat* mutant

The association with the parthenocarpic phenotype was the first evidence that SlHB15 is involved in the control of fruit set (Clepet et al., 2021; this work). Accordingly, the *Arabidopsis meristem enlargement 1* (*men1*) mutant, in which MIR166a is activated by the insertion of the CaMV35S enhancer leading to a drastic reduction of *HB15* mRNA level, when pollinated with wild-type pollen, produced fruits with no seeds (Kim et al., 2005). Like the *pat* mutant, *men1* exhibited pleiotropic alterations in floral and leaf morphology. Other homeobox TFs were involved in the control of ovary growth, such as the tetratricopeptide repeat protein *SlTPR1* (Lin et al., 2008), or *BELL1* (*BEL1*)-like genes whose products regulate ovule development and post-pollination ovary changes in *Phalaenopsis* (Nadeau et al., 1996) and apple (Dong et al., 2000).

The *pat* parthenocarpic phenotype is associated with aberrations in ovule integument growth (Mazzucato et al., 1998). In *pat* ovaries and fruits, aberrant ovules coexist with normally developed ones. Previous studies showed that the frequency of aberrant ovules in *pat* is positively correlated with the penetrance and expressivity of the parthenocarpic phenotype (Mazzucato et al., 1999; **Supplementary Figure 7**). Thus, a regulatory role of the ovule to repress ovary development before pollination should be postulated. Defects in ovule integuments were also associated with the production of seedless fruits in sugar apple (Lora et al., 2011), sweet pepper (Tiwari et al., 2011), eggplant (Takisawa et al., 2012), and other tomato parthenocarpic systems (Wang et al., 2009; da Silva et al., 2017; Rojas-Gracia et al., 2017; Gupta et al., 2021). Moreover, upregulation of the tomato ortholog of *Aintegumenta*, *SlANT*, was common to *pat* (Ruiu et al., 2015) and *agl6* (Gupta et al., 2021) parthenocarpic mutants. The latter authors also describe several DOF-like genes, which are upregulated in mutant ovules and are predominantly expressed in the integument and funiculus. Among them, *Solyc06g075370*, corresponding to DOF6 in *Arabidopsis* is strongly expressed in the funiculus and is functionally related to HD-Zip III genes (Miyashima et al., 2019). Thus, SlHB15 regulate ovule integument and vasculature development, exerting a control on fruit set. As the HB15 transcript is retained in aberrant ovules in the mutant, in contrast to a lower level detected in the entire ovary, a defect in the transport of this repressing factor may be hypothesized to explain the *pat* phenotype. All this evidence suggests that proper ovule development is essential to control ovary growth before anthesis; disruption of such control releases ovary growth and yields parthenocarpic seedless fruits.

### 4.4 The HD Zip III effect on fruit set is mediated by auxin

Altogether, the data collected suggest that SlHB15 is an inhibitor of IAA signaling (Clepet et al., 2021; da Silva and Nogueira, 2021), in agreement with phenotypes of the *pat* mutant that involve increased IAA response, such as parthenocarpy, increased root development and higher regenerative capacity (Habashy et al., 2004). Also, the feminization of stamens occurring in *pat* (Mazzucato et al., 1998) is in line with an IAA response (Sawhney and Shukla, 1994). This action can be mediated either directly or through ARFs or by affecting auxin transport through *PIN-FORMED* (*PIN*) genes. PIN proteins control the dynamic changes in auxin flux and maxima, regulating the transcription of ARFs, that activate and/or repress downstream target genes. Mutations in members of the *ARF* gene family in *Arabidopsis* and tomato resulted in fruit set in the absence of pollination and fertilization (Goetz et al., 2007; Gorguet et al., 2008; de Jong et al., 2009). Indeed, physical interaction between SlHB15, SlPIN4 and SlARF7 has been shown and functional interaction between SlHB15 and PIN1 is corroborated by expression in the same ovule tissues of the respective genes (Mounet et al., 2012; Clepet et al., 2021; this work).

Consistently, we propose a model that explains the function of SlHB15 in the molecular pathway of fruit set by supporting the action of SlARF7 (a transcriptional activator of “auxin response attenuating genes,” de Jong et al., 2009). Thus, the SlHB15 function upstream to this cascade might consists in the positive regulation of *ARF7*. After pollination, the *KAN* gene family, acting antagonistically with HD-Zip III TFs, would downregulate *SlHB15* and consequently *SlARF7*, giving rise to the fruit set. In the *pat* mutant, the loss of SlHB15 function hampers the transcription of *SlARF7* (Ruiu et al., 2015; Clepet et al., 2021) and the absence of ARF7 prevents the activation of the “auxin response attenuating genes” and consequently ovary repression.

### 4.5 The *pat* mutant shares vegetative and reproductive phenotypes with *HB15 Arabidopsis* mutants

Several vegetative phenotypes of mutants affecting *HB15*, such as *cna-1*, *cna-2*, *icu4-1* and *high shoot-organogenic capacity (hoc)*, have been described in *Arabidopsis* (Green et al., 2005; Prigge et al., 2005; Ochando et al., 2006; Kelley et al., 2009; Duclercq et al., 2011), but reproductive traits of such mutants were not observed in detail. Whereas loss-of-function mutations rarely showed evident phenotypes due to the high-functional redundancy, those showing gain-of-function usually showed developmental phenotypes. The *hoc* T-DNA mutant has one of the most drastic phenotypes, including the ability to regenerate whole plants *in vitro* without added phytohormones (Catterou et al., 2002). Similarly, *pat* showed a higher regeneration index than the WT (Habashy et al., 2004), also in parallel with Aux/IAA9 parthenocarpic knockouts (Wang et al., 2005).

The characterization of vegetative traits in both *cna-1* and *icu4-1* single mutants highlighted alterations like those displayed by *pat* in tomato. These findings indicated that *HB15* alone, if mutated, could affect the plant stature (*cna-1*) and cotyledon development (*icu4-1*). A typical *pat* vegetative defect is the occurrence of seedlings with extra cotyledons or cotyledons with altered morphology (Olimpieri et al., 2007). Supernumerary or defective cotyledons have been reported in mutants altered in polar IAA transport, such as *pinoid* (*pid*) in *Arabidopsis* (Bennett et al., 1995) and *polycotyledon* (*poc*) in tomato (Al-Hammadi et al., 2003). In parallel with *pat*, defects in cotyledon number and structure have also been reported in tomato genotypes affected in fruit set (Wang et al., 2005), showing that perturbations described at the fruit set level are reflected during embryogenesis. In parallel, higher order knockouts of HD-Zip III genes reveal redundant functions in embryo and cotyledon patterning in *Arabidopsis* (Prigge et al., 2005) and tricotyledonary seedlings were evidenced in the *icu4-1* gain-of-function mutant (Ochando et al., 2006; this work). Supernumerary cotyledons were found in plants overexpressing HD-Zip III members due to the reduction of *miR165* (Jia et al., 2015). Altogether these data show that HD-Zip III TFs are responsible for proper IAA distribution-mediated meristem regulation also at the vegetative level.

In parallel with the *pat* mutant, *Arabidopsis HB15* single mutants showed defects in ovule and seed development, which were paralleled by fertilization-independent ovary enlargement. This observation supports the idea that, although several HD-Zip III members are involved in the differentiation of ovule integuments, HB15 could represent the main regulator of this trait (Kelley et al., 2009), following the knowledge that the HB15/HB8 subclade shows less redundancy than REV/PHB/PHV (Prigge et al., 2005). Moreover, the results indicate that both loss- and gain-of-function mutants of this gene could lead to the production of aberrant ovules, a phenotype that is positively associated with a capacity for parthenocarpic silique development. Parallel behavior of *HB15* mutants in tomato and *Arabidopsis* indicate a conservation on mechanisms for fruit set control in species with very different fruit type.

## Data availability statement

The original contributions presented in the study are included in the article/**Supplementary Material**. Further inquiries can be directed to the corresponding author.

## Author contributions

MEP: Formal analysis, Investigation, Methodology, Project administration, Writing – original draft, Writing – review & editing. FR: Formal analysis, Investigation, Methodology, Writing – original draft, Writing – review & editing. LS: Formal analysis, Investigation, Methodology, Writing – original draft, Writing – review & editing. SP: Investigation, Writing – review & editing. CM: Investigation, Writing – review & editing. SM: Methodology, Writing – review & editing. LC: Conceptualization, Methodology, Writing – review & editing. GPS: Conceptualization, Funding acquisition, Project administration, Resources, Writing – review & editing. AG: Conceptualization, Funding acquisition, Methodology, Project administration, Resources, Writing – review & editing. AM: Conceptualization, Formal analysis, Funding acquisition, Investigation, Methodology, Project administration, Resources, Writing – original draft, Writing – review & editing.
