# Supplementary material for: Genetic and molecular mechanisms underlying the parthenocarpic fruit mutation in tomato
Source: Front Plant Sci. 2024 Mar 27;15:1329949. doi: 10.3389/fpls.2024.1329949 (PMC11004453; doi:10.3389/fpls.2024.1329949)
Supplement: Supplementary file 1 [file DataSheet_1.pdf]

Supplementary Material

Supplementary Data

1 Development of new markers in the *Pat* genetic window using tomato/Arabidopsis microsynteny

To find new markers associated to the *Pat* genetic window, we exploited the microsynteny existing between the Arabidopsis and tomato genomes (Fulton et al., 2002). Two unlinked Arabidopsis BACs, T2O4 (Chr3) and F19K16 (Chr1), matching respectively the Conserved Ortholog Sequence (COS) markers T1143 and T0796, were selected at TAIR (The Arabidopsis Information Resources; www.arabidopsis.org). A total of 47 Arabidopsis gene sequences, 24 surrounding T1143 on BAC T2O4 (T1 to T24) and 23 surrounding T0796 on BAC F19K16 (F1 to F23) were used to identify ortholog genes in the tomato genome using BLAST2 and searching the Solanaceae Genomics Network (SGN; www.sgn.cornell.edu) database.

Twenty-one tomato sequences with univocal orthology to the Arabidopsis genes were identified and portions of these sequences amplified in the parental lines (not shown). PCR was carried out in a total volume of 25 µl, using 50 ng of DNA, 2.5 µl of 10 x PCR buffer, 2 µl of 10 mM dNTPs, 1.5 µl of 25 mM $MgCl_2$, 50 pmol of each of the two primers and 1 U Taq DNA polymerase (Pharmacia Biotech, San Francisco, CA). After a denaturation step of 95°C for 4 min, amplification was carried out for 30 cycles of 95°C for 1 min, 1 min at the annealing temperature suggested by the primer synthesis service and 72°C for 2 min, followed by 72°C for 7 min. PCR products were resolved by electrophoresis on 1% (w/v) agarose gels stained with ethidium bromide. Nine of these loci were confirmed on Chr3 by PCR on Alien Substitution Lines (ASL) or Introgression Lines (IL; Eshed and Zamir 1995). In addition, markers SSR320, C2_At1g55170 and C2_At2g42110, mapping within the *Pat* genetic window according to the EXPEN2000 published map (https://solgenomics.net) were also tested in the segregating population (Supplementary Table 1).

PCR amplification for these 12 loci (Supplementary Table 1) gave In/Del polymorphism between parents in five cases. For those PCR products of similar size in the two parents, cleaved amplified polymorphic sequences (CAPS) polymorphisms were searched using several 4- and 6-cutter restriction enzymes. The CAPS technique was performed by digesting 10 µl of the PCR reaction with 5 U of the appropriate enzyme and restriction fragments were resolved onto 2.0% (w/v) agarose gels. Finally, six new markers, T17, T18, T20, T21, F5, and C2_At2g42110 were mapped within the region flanked by T0796 and T1143, thus refining the *Pat* window to 0.19 cM.

2 Chromosome walking towards the *Pat* locus

The COS markers T0796 and T1143 matched several tomato Bacterial Artificial Chromosome (BAC) clones belonging to two unlinked contigs, CTG6859 and CTG10242 (Supplementary Data Figure 1). BAC singletons whose end sequences had a high identity value (>95%) with either the end sequences or the complete sequences of BACs belonging to the contigs 6859 or 10242 were identified using BLAST2. The recombinant BAC clones, constructed with the *Hind*III/*Mbo*I/*Eco*RI-

pBeloBAC11 vector and hosted in DH10B *Escherichia coli* strains, were grown in 10 ml of LB medium with 12 µg/ml of chloramphenicol as selective antibiotic. BAC DNA was extracted and purified using the QIAGEN Plasmid Midi Kit (QIAGEN Inc., Valencia, CA, USA). Bacterial lysates were cleared by centrifugation. The cleared lysates were then loaded onto an anion-exchange tip where the BAC clone DNA selectively bound under appropriate low-salt and pH conditions. RNA, proteins, metabolites, and other low-molecular-weight impurities were removed by a medium-salt wash, and ultrapure plasmid DNA was eluted in high-salt buffer. The DNA was concentrated and desalted by isopropanol precipitation and collected by centrifugation. After a wash with 500 µl of 70% ethanol, the vacuum dried DNA pellets were dissolved in 50 µl of sterile water. DNA concentration was determined by spectrophotometric measure at 260 nm and adjusted at 200 ng/µl. Samples were stored at –20° C. Amplifications on the BAC clones were conducted in the same way as for the plant DNA.

3 Physical vs genetic mapping

COS F5, mapped between T0796 and the *Pat* locus, matched BACs Hba210F21, Hba043F15 and Hba0177L08 in CTG6859. The T7 end sequence of BAC MboI0001K18 overlapped with the Sp6 sequence of BAC Hba210F21 and its opposite end could be mapped within the window cosegregating with *Pat*. On the other side, the T1143 sequence matched few BACs of CTG10242, among which Hba166B19. The Sp6 end of BAC Hba0025G09 overlapped with the T7 sequence of BAC Hba166B19, thus orienting Hba0025G09 within the target region. End sequences of BACs confirmed to be located on the *Pat* locus region through markers matching, were used to develop new molecular markers. Specific primers were designed based on the published sequences at SGN (not shown). Once a polymorphism was detected, its map position was validated by screening a set of *S. pennellii* IL (Eshed and Zamir1995) and the recombinant plants in the mapping populations. However, the two chromosome walking paths could not overlap, as COS markers T17, T18, T20 and T21 did not match any BAC. Finally, markers T18, C2_At2g42110 and the T7 ends of Hba0228N05 and MboI0001K18 all cosegregated with the *Pat* locus. The physical resolution of the *Pat* genetic window was possible once the tomato genome sequence became available (The Tomato Genome Consortium, 2012).

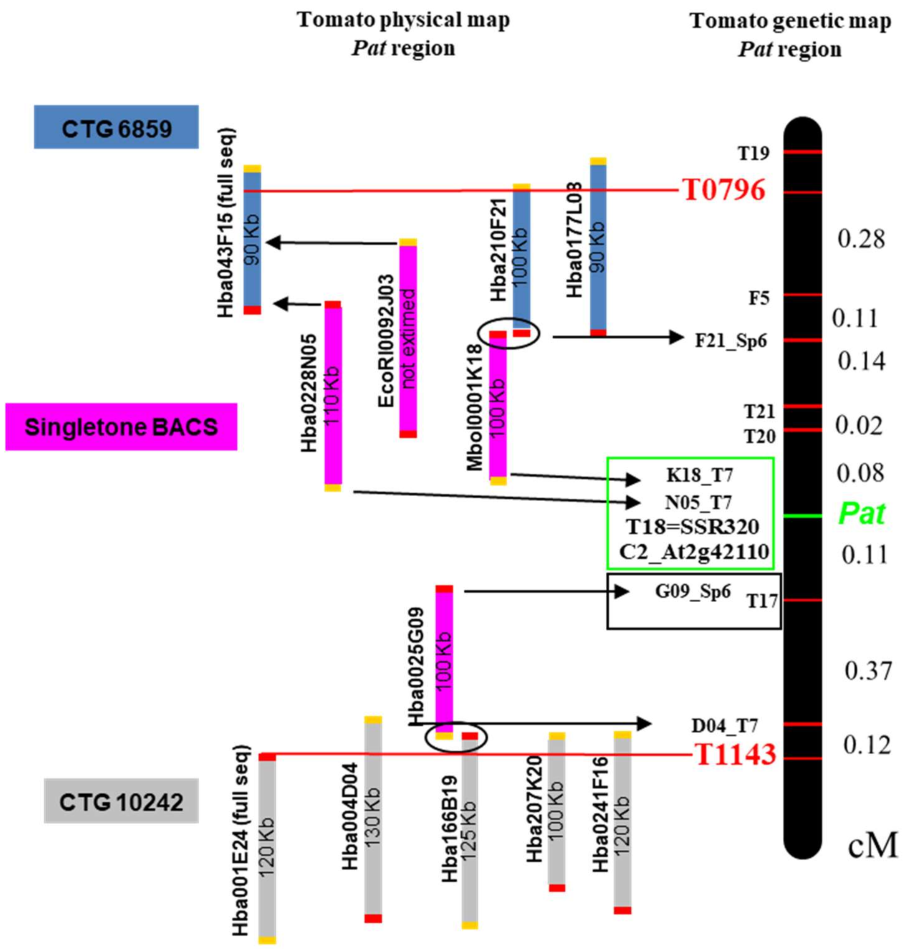

Supplementary Data Figure 1. Fine mapping of the *Pat* gene and integration of the genetic and the physical map. Relative position of new markers developed and mapped within the T0796-T1143 *Pat* window and attempts of chromosome walking through BAC ends matching analysis. BACs have the Sp6 end sequence in red and the T7 end sequence in yellow.
