# Supplementary material for: Genetic and molecular mechanisms underlying the parthenocarpic fruit mutation in tomato
Source: Front Plant Sci. 2024 Mar 27;15:1329949. doi: 10.3389/fpls.2024.1329949 (PMC11004453; doi:10.3389/fpls.2024.1329949)
Supplement: Supplementary file 2 [file Image_1.pdf]

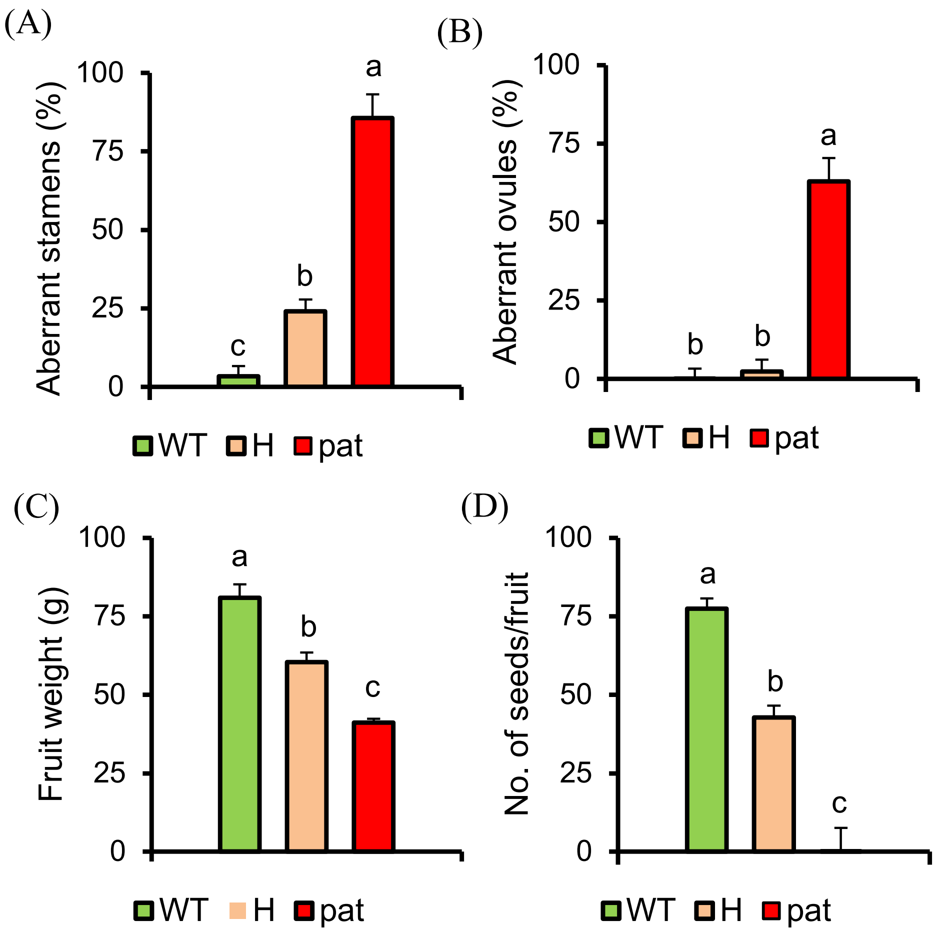

Supplementary Figure 1. Phenotyping of plants segregating for the *SlHB15^pat^* allele. Phenotypes of plants homozygous for the wild type (WT) or the *pat* mutant (*pat*) allele or heterozygous (H) for the frequency of aberrant **(A)** stamens and **(B)** ovules, **(C)** for the fruit weight and **(D)** for the mean number of seeds per fruit. Data are means of at least ten flowers in (A) and (B) and of at least ten fruits in (C) and (D). Means indicated by different lowercase letters are statistically different for $P \leq 0.05$ after Duncan's multiple range test.

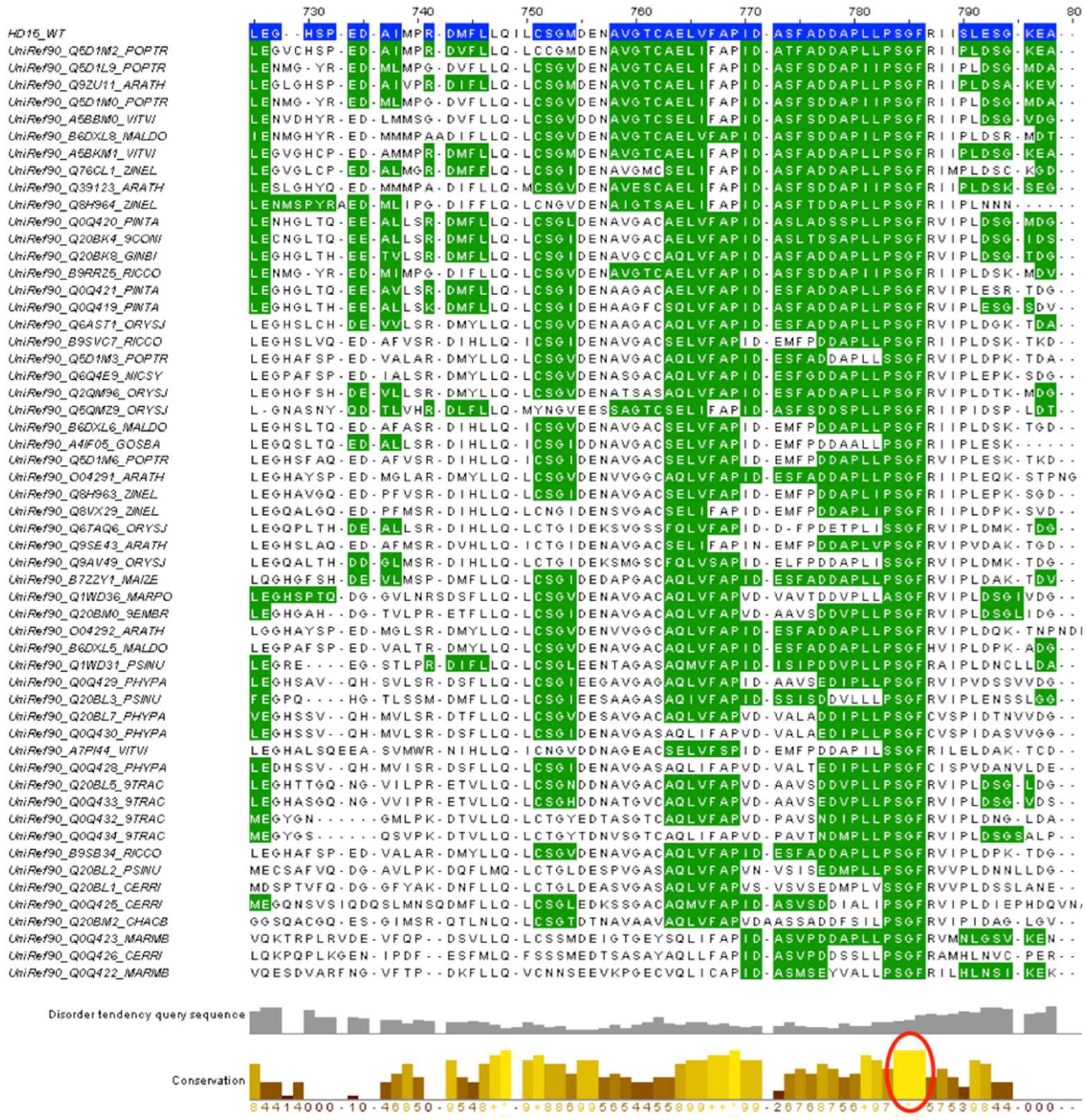

Supplementary Figure 2. Alignment of the region mutated in *SlHB15*^*pat*^ (in blue) with 55 ortholog proteins (ELM server; http://elm.eu.org/). Analysis of homology of 55 higher plants *HB15*-like sequences in the region spanning the purposed *pat* mutation. Circled is a cluster of four highly conserved amino-acids, containing G583.

(A)

(B)

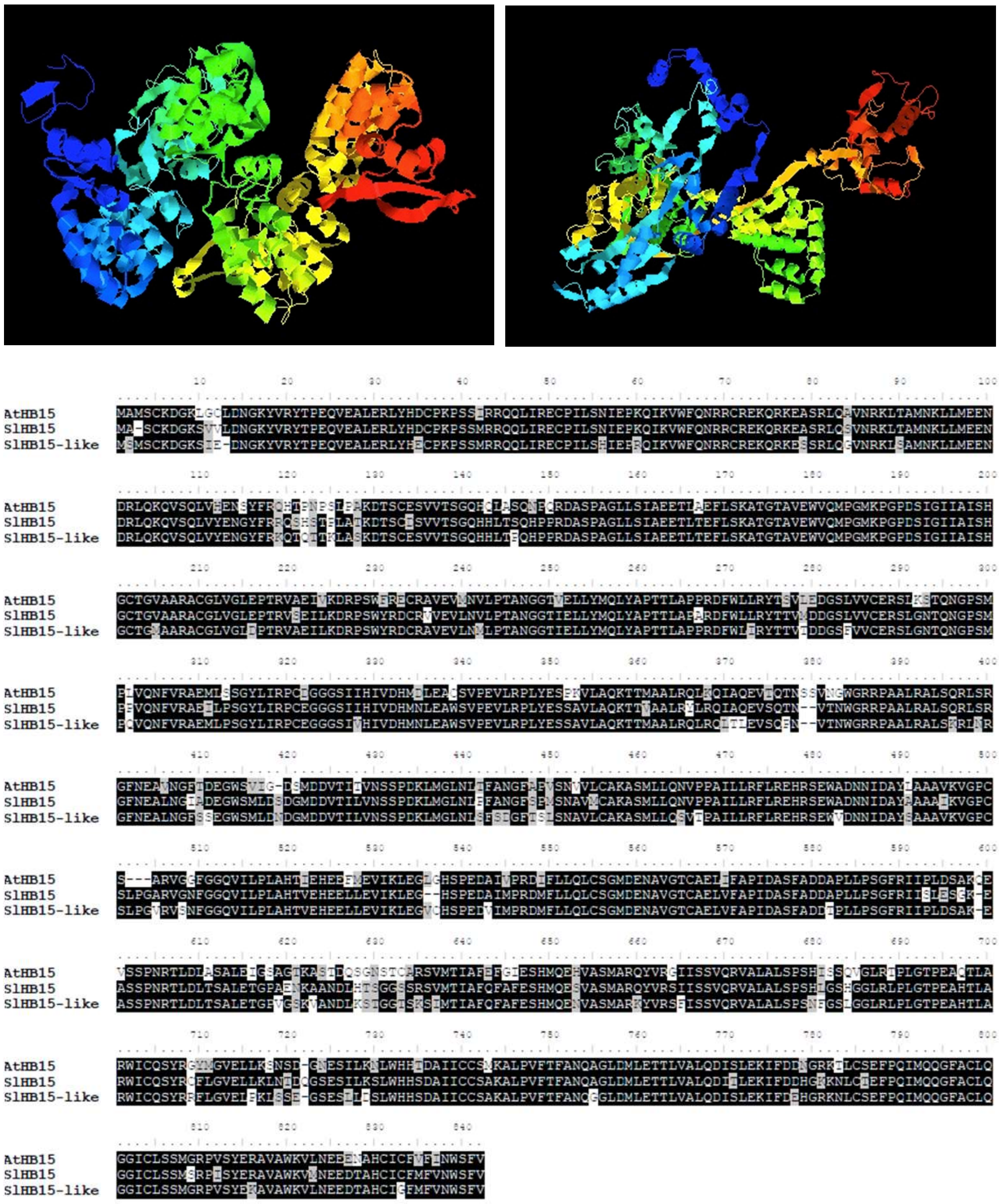

Supplementary Figure 3. Structural prediction of the *pat* mutation effect and alignment of HD-Zip III proteins in tomato and Arabidopsis. (A) I-TASSER prediction of the 3D structure models of the WT and *pat* mutant variant of the SlHB15 protein. **(B)** Alignment of the AtHB15, SlHB15 and SlHB15-like protein sequences (amino acid identity and similarity are shadowed in black and grey respectively)

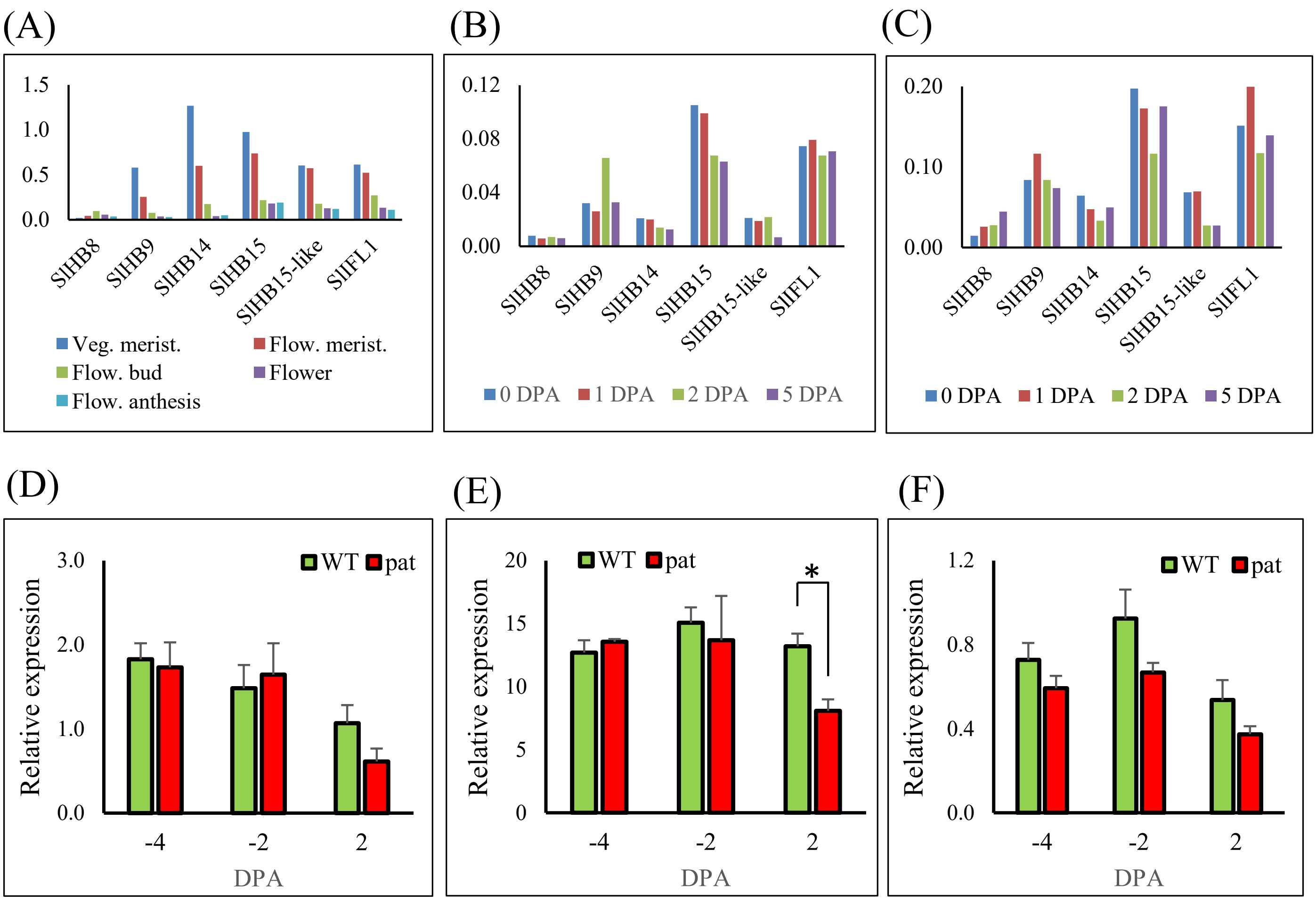

Supplementary Figure 4. Expression data of HD-Zip III tomato genes retrieved from the Tomexpress database (http://tomexpress.toulouse.inra.fr/) and from previous analysis (Ruiu et al., 2015). Expression of the six HD-Zip III tomato genes in **(A)** vegetative and reproductive meristems and in whole flower buds and flowers, **(B)** ovules and **(C)** pericarp at different days post anthesis (DPA). Expression in WT and *pat* ovaries at -4, -2 and 2 DPA of **(D)** *HB15-like* after qRT-PCR, and **(E)** *SlHB15* and **(F)** *SlHB15-like* as retrieved from published microarray data (Ruiu et al., 2015). * indicates significant difference between genotypes within tissue for $P \leq 0.05$. Data in (A)-(C) are normalized mean counts per base.

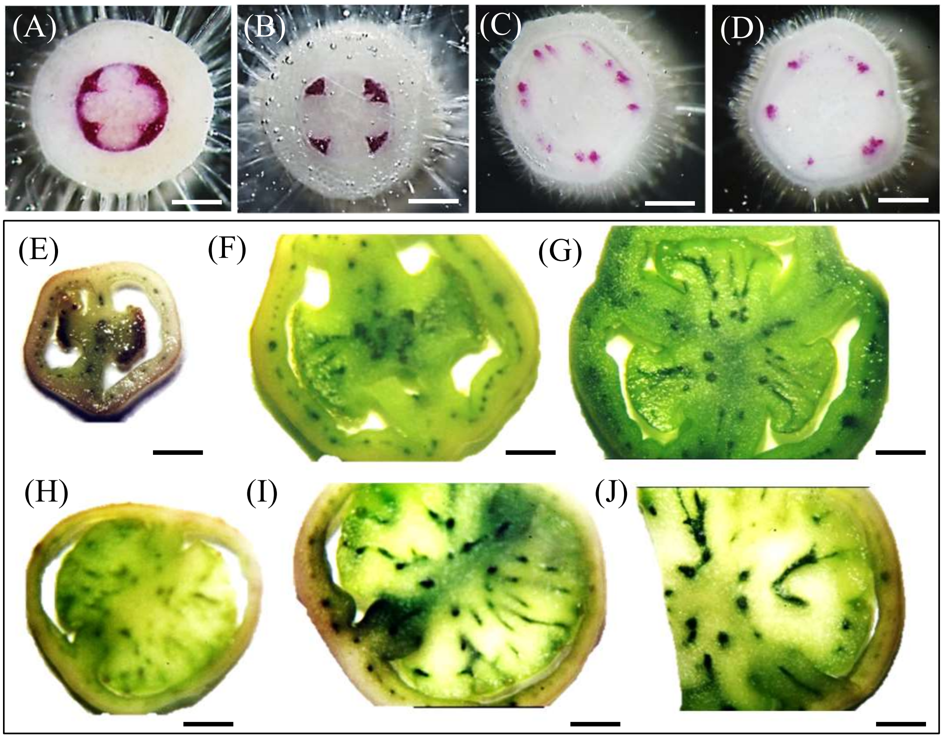

Supplementary Figure 5. Analysis of vasculature development in seedlings and fruits of the WT and of the *pat* mutant. Arrangement of the vasculature in **(A)** the hypocotyl and **(C)** the epicotyl of WT and **(B, D)** *pat* 30-d-old plantlets, respectively. Auxin distribution detected through an IAA-sensitive reporter construct in fruitlet and fruit sections of the WT at **(E)** 6 and **(F)** 11 days post anthesis and **(G)** at the mature green stage; **(H)** to **(L)** sections of *pat* mutant fruits at the same stages. Scale bar is 1mm in (A)-(E), 2 mm in (F) and (H), 3 mm in (I), and 5 mm in (G) and (J).

(A)

Guide-18 score: 98

postion: SL2.50ch03:-69144152

guide sequence: GCAGGACTAGCATCCCTCGGCGG

Sequence	Score	MMs	Locus	Gene	Region
TCTGGACTAGCATCCCTGGGAGG	0.7	3MMs	SL2.50ch04:-60066211	Solyc04g074040.2	cds
GCAGGACTAGCATCTCTTGGAGG	0.3	2MMs	SL2.50ch12:+38532328	Solyc12g044410.1	cds
TCAGGACAAGAATCCCTCAGGGG	0.3	4MMs	SL2.50ch03:-70713768	Solyc03g124010.2	cds
GCAGGGCTTGCATCCCTTGGTGG	0.2	3MMs	SL2.50ch08:+55211212	Solyc08g066500.2	cds
GCAGGACTATTATACTTCGGAGG	0.0	4MMs	SL2.50ch01:+88626632	Solyc01g097980.2	cds

(B)

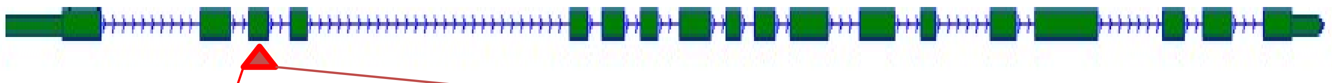

```
WT    (0)   GCATCCGCCG-AGGGATGCTAGTCCTGCAGGACTTTTGTCC
GE-1  (-5)  GCATCCGCCG------TGCTAGTCCTGCAGGACTTTTGTCC
GE-2a (+1)  GCATCCGCCGAAGGGATGCTAGTCCTGCAGGACTTTTGTCC
GE-2b (-7)  GCATCCGCCG--------CTAGTCCTGCAGGACTTTTGTCC
```

Supplementary Figure 6. Generation of gRNA and different breakpoints of plants edited at the *SlHB15* gene. (A) CRISPR-P output showing the on-target score for gRNA18, its position in the reference tomato genome (SL2.5 version) and sequence, including the PAM region (highlighted in green). In addition, it shows the sequences of five putative off-target loci with their relative score, number of mismatches (in red), position in the reference tomato genome, and the name of the gene containing the putative off-target site in the coding region. **(B)** Position of the gRNA18 locus in the third exon of *SlHB15* (*red arrowhead*) and type of DNA lesions generated in two studied SlHB15^{CRISPR} T_0 plants, including a homozygous 5-bp deletion (*dashed sequence*) in GE-1, and a single-nucleotide insertion (*bold blue letter*) and a 7-bp deletion in GE-2 (allele GE-2a and GE-2b, respectively). The reverse complement of the sgRNA target (red) and the PAM site (*bold black*) are shown in the WT sequence.

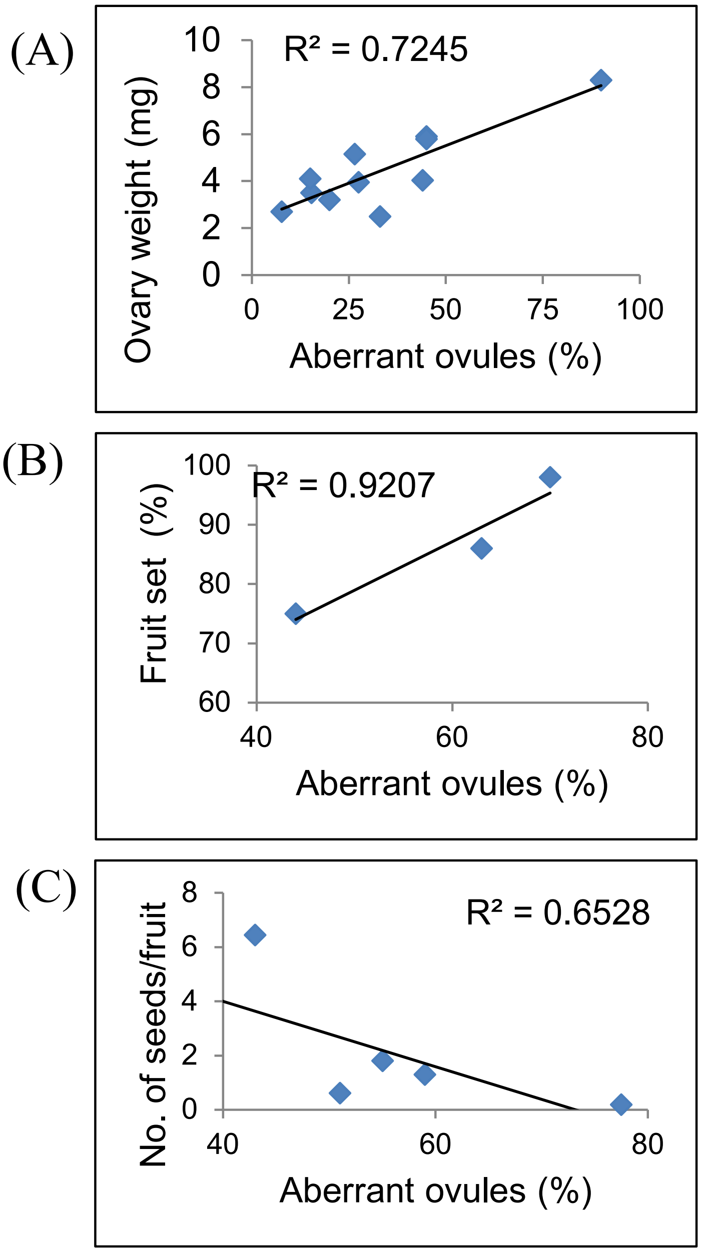

Supplementary Figure 7. Relationship between the frequency of aberrant ovules and the expression of parthenocarpy in the *pat* mutant. Linear regression between the frequency of aberrant ovules and **(A)** the ovary weight at anthesis, **(B)** the percentage of fruit set and **(C)** the number of seeds per fruit. All data are retrieved from published research cited in the text.
